# Sja-let-7 suppresses the development of liver fibrosis via *Schistosoma japonicum* extracellular vesicles

**DOI:** 10.1371/journal.ppat.1012153

**Published:** 2024-04-10

**Authors:** Haoran Zhong, Bowen Dong, Danlin Zhu, Zhiqiang Fu, Jinming Liu, Yamei Jin

**Affiliations:** 1 National Reference Laboratory for Animal Schistosomiasis, Shanghai Veterinary Research Institute, Chinese Academy of Agricultural Sciences, Shanghai, P.R. China; 2 Key Laboratory of Animal Parasitology of Ministry of Agriculture and Rural Affairs, Shanghai Veterinary Research Institute, Chinese Academy of Agricultural Sciences, Shanghai, P.R. China; Aarhus University Department of Clinical Medicine: Aarhus Universitet Institut for Klinisk Medicin, DENMARK

## Abstract

Schistosomiasis is a fatal zoonotic parasitic disease that also threatens human health. The main pathological features of schistosomiasis are granulomatous inflammation and subsequent liver fibrosis, which is a complex, chronic, and progressive disease. Extracellular vesicles (EVs) derived from schistosome eggs are broadly involved in host-parasite communication and act as important contributors to schistosome-induced liver fibrosis. However, it remains unclear whether substances secreted by the EVs of *Schistosoma japonicum*, a long-term parasitic “partner” in the hepatic portal vein of the host, also participate in liver fibrosis. Here, we report that EVs derived from *S*. *japonicum* worms attenuated liver fibrosis by delivering sja-let-7 into hepatic stellate cells (HSCs). Mechanistically, activation of HSCs was reduced by targeting collagen type I alpha 2 chain (Col1α2) and downregulation of the TGF-β/Smad signaling pathway both *in vivo* and *in vitro*. Overall, these results contribute to further understanding of the molecular mechanisms underlying host-parasite interactions and identified the sja-let-7/Col1α2/TGF-β/Smad axis as a potential target for treatment of schistosomiasis-related liver fibrosis.

## Author summary

Schistosomiasis is a neglected parasitic disease that affects over 250 million people in tropical and subtropical regions worldwide. As common pathogenic species, the eggs of *Schistosoma japonicum* and *S*. *mansoni* cause hepatic schistosomiasis, which can lead to granulomatous inflammation and subsequent liver fibrosis. Hence, elucidation of the mechanisms underlying liver fibrosis and host-parasite crosstalk is essential to inhibiting the progression of schistosomiasis. Accumulating evidence supports the pivotal role of schistosome egg-derived extracellular vesicles (EVs) in the onset and progression of schistosomiasis. However, the potential involvement of schistosome worm-derived EVs in the activation and modulation of hepatic stellate cells (HSCs) in hepatic schistosomiasis remains largely unknown. The results of this study demonstrate that the release of sja-let-7 by *S*. *japonicum* worm-derived EVs reduced activation of HSCs by targeting Col1α2 and further attenuated the progression of liver fibrosis by mediating the TGF-β/Smad signaling pathway. These findings provide evidence for the application of worm-derived substances and the sja-let-7/Col1α2/TGF-β/Smad axis as a novel therapeutic target for treatment of liver fibrosis.

## Introduction

Schistosomiasis is a neglected tropical disease caused by infection with blood flukes of the genus *Schistosoma* that affects more than 250 million people each year mainly in tropical and subtropical regions of developing countries worldwide, accounting for an estimated 1.4–3.3 million disability-adjusted life years annually [1,2]. The average survival of schistosomes in human hosts is 3–10 years and reportedly up to 40 years in extreme cases [3]. Adult schistosomes typically exist in worm pairs. During mating, the male-female pairs migrate to the mesenteric vessels of the host, where the females produce eggs, which are responsible for the spread and primary pathology of schistosomiasis [4]. Mature female *S*. *japonicum* and *S*. *mansoni* worms produce hundreds of eggs per day and most are deposited in the liver of the host, which leads to granulomatous inflammation and eventual fibrosis due to the host immune response. Schistosomiasis is particularly threatening to human health because of the lack of effective strategies for prevention and treatment [5].

Hepatic stellate cells (HSCs) are the principal collagen-producing cells in the liver and play a crucial role in schistosome-induced fibrogenesis [6]. In response to liver damage, HSCs differentiate into myofibroblasts, which are characterized by synthesis of fibril-forming (type I and III) collagen and the extracellular matrix protein α-smooth muscle actin (α-SMA) [7]. Therefore, it is particularly important to elucidate the roles and mechanisms of HSCs in schistosome-induced hepatic fibrosis.

The intricate relationship of *S*. *japonicum* with the human host enables the parasite to survive and evade host immunity. Extracellular vesicles (EVs) are small vesicles (diameter, ~100 nm) generated by all cells that transport various molecules, such as nucleic acids, proteins, lipids, and metabolites, and facilitate parasite–host crosstalk [8–10]. Schistosome eggs, the central part of granulomas, mediate activation of HSCs through secretion of schistosome miRNAs, such as sja-miR-1 [11], sja-miR-71a [12], sja-miR-2162 [13] and a novel miRNA-33 [14]. Each of these miRNAs plays a regulatory role through a unique mechanism and is involved in the dynamics of liver fibrosis. Notably, sja-miR-71a exhibits anti-fibrotic effects, suggesting that schistosomes secrete anti-pathological substances to prolong the life of the host as long as possible, resulting in chronic progressive disease, such as liver fibrosis.

However, comparisons of *Sj*-miRNAs in the EVs derived from the *S*. *japonicum* worm [15], egg [16] and primary HSCs of infected mice [13] identified four *Sj*-miRNAs transported to primary HSCs via worm-derived EVs, suggesting that in addition to the schistosome egg, the worm might also be involved in the process of liver fibrosis. However, the potential contributions of *Sj*-miRNAs from worm-derived EVs remain largely unknown.

The results of this study revealed that sja-let-7 released mostly by adult worm-derived vesicles reduced activation of HSCs by targeting Col1α2 and further attenuated progression of liver fibrosis by mediating the TGF-β/Smad signaling pathway. These findings highlight a worm-induced fibrotic regulatory process that expands current understanding of host-parasite interactions and also provides a potential target for treatment of liver fibrosis.

## Results

### Comparisons of *Sj*-miRNAs from the EVs of *S*. *japonicum* worms and eggs with primary HSCs of infected mice

A previous study identified eight *Sj*-miRNAs from primary HSCs of infected mice by RNA sequencing [13], which are reportedly transported to the cytosol of HSCs via *Sj*EVs [17]. In the present study, *Sj*-miRNAs derived from the EVs of *S*. *japonicum* worms [15] and eggs [16] were compared to the eight *Sj*-miRNAs previously identified in the primary HSCs of infected mice (S1 Table). A Venn diagram illustrated that four *Sj*-miRNAs (sja-bantam, sja-miR-2a, sja-miR-10, and sja-miR-2162) were likely transported to HSCs via egg-derived EVs, while the other four *Sj*-miRNAs (sja-let-7, sja-miR-1, sja-miR-125b, and sja-miR-190) might be transported to HSCs via worm-derived EVs (S1 Fig). Comparisons of *Sj*-miRNAs from the EVs of *S*. *japonicum* worms and eggs with primary HSCs of infected mice suggest that in addition to schistosome eggs, worms may also be involved in the process of liver fibrosis with HSCs as the main effector cells.

### Sja-let-7 derived from *S*. *japonicum* worms is involved in the activation of HSCs

**Fig 1:**
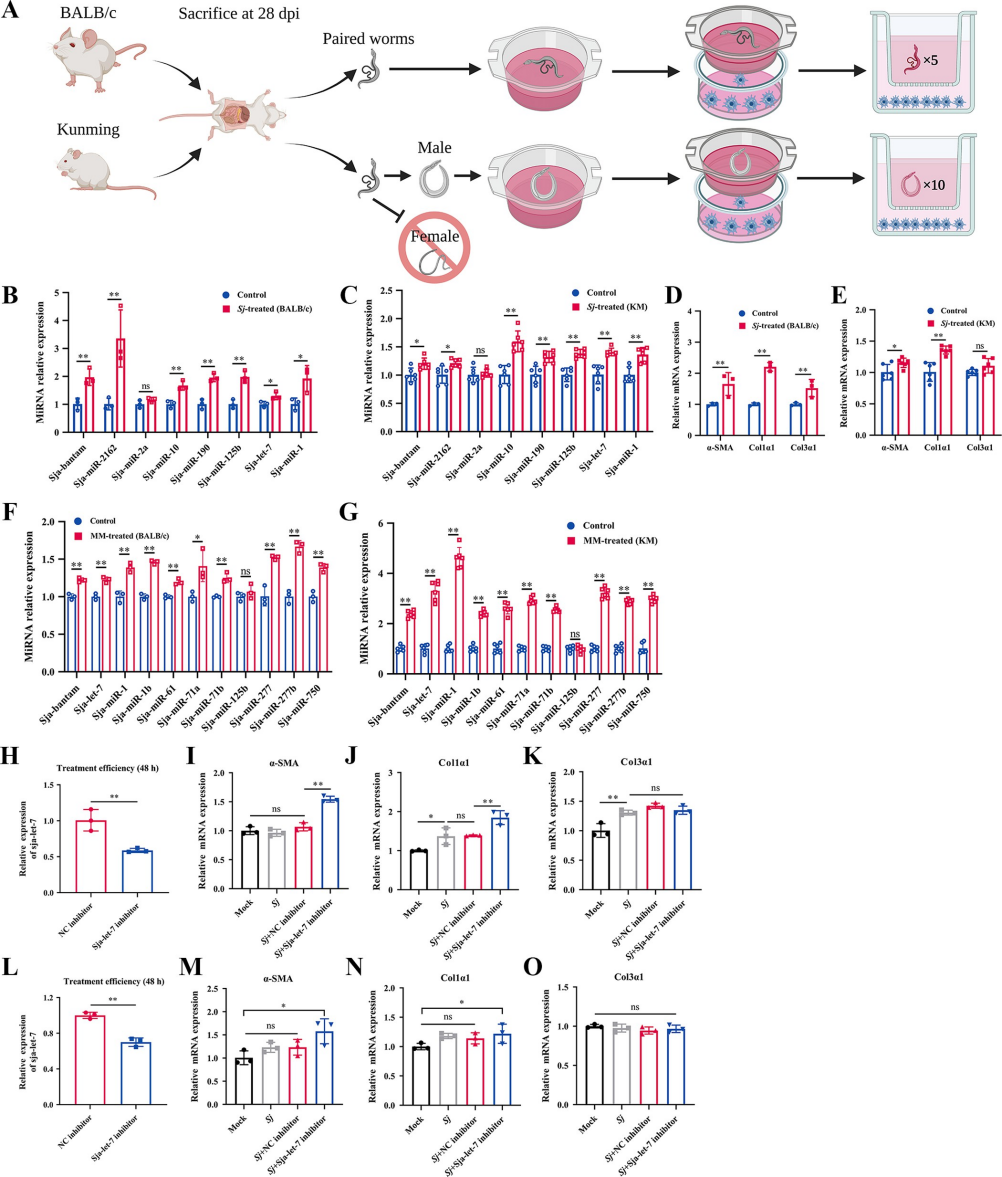
Sja-let-7 derived from *S*. *japonicum* worms involves in the activation of HSCs. (A) Workflow of transwell systems establishment. (B-C) Detection of 8 *Sj*-miRNAs in the LX-2 cells from the transwell system composed by worms coming from BALB/c mice (n = 3) and KM mice (n = 6) (D-E) Detection of α-SMA, Col1α1 and Col3α1 mRNA expression of the LX-2 cells from the transwell system composed by worms coming from BALB/c mice (n = 3) and KM mice (n = 6). (F-G) Detection of 11 *Sj*-miRNAs in the LX-2 cells from the transwell system composed by MM worms coming from BALB/c mice (n = 3) and KM mice (n = 6). (H-K) Treatment efficiency analysis and detection of α-SMA, Col1α1 and Col3α1 mRNA expression towards LX-2 cells from the transwell system composed by worms coming from BALB/c mice (n = 3). (L-O) Treatment efficiency analysis and detection of α-SMA, Col1α1 and Col3α1 mRNA expression towards LX-2 cells from the transwell system composed by worms coming from KM mice (n = 3). All graph data are expressed as the mean ± SD of at least three biological replicates per group. *P< 0.05, **P< 0.01, ns, not significant. Abbreviation: *Sj*: *S*. *japonicum*; MM: mated male. Panel A was created with Biorender.com.

Adult *S*. *japonicum* worms can survive in the portal vein of the host for decades [2]. A transwell coculture system was used to simulate the environment of the portal vein to determine whether molecules of worm-derived EVs are involved in activation of HSCs. In the present study, adult paired worms from two naturally permissive hosts (BALB/c and Kunming (KM) mice) were used to strengthen the generality of the conclusions (Fig 1A). Following coculture for 48 h, the eight *Sj*-miRNAs mentioned above [13] were selected and measured by quantitative real-time polymerase chain reaction (qPCR) to investigate whether worms directly secrete substances into HSCs. The results showed that sja-let-7, along with six other *Sj*-miRNAs, were significantly increased in HSCs from both transwell systems, while sja-miR-2a was not detected (Fig 1B and 1C). In addition, qPCR analysis revealed that the mRNA levels of two markers of fibrosis (α-SMA and Col1α1) were significantly increased in HSCs from both transwell systems, while Col3α1 mRNA levels were only up-regulated in the transwell systems containing worms from BALB/c mice (Fig 1D and 1E). Since adult paired worms can also lay eggs in the upper wells of the transwell systems, to exclude the effects of unknown substances from eggs, mated male (MM) worms from BALB/c and KM mice were separated *in vitro* and cocultured with HSCs for 48 h in both transwell systems (Fig 1A). Then, 11 previously reported *Sj*-miRNAs, including sja-let-7, from MM derived-EVs were selected for further experiments [18]. The presence of the 10 *Sj*-miRNAs out of 11 in HSCs, including sja-let-7, was verified by qPCR, while sja-miR-125b were undetectable in both transwell systems (Fig 1F and 1G).

To further explore whether sja-let-7 is involved in activation of HSCs, paired adult worms from BALB/c and KM mice were treated with sja-let-7 and negative control (NC) inhibitors, respectively. After coculture for 48 h, qPCR analysis was conducted to assess the uptake efficiency of the HSCs (Fig 1H–1O). The mRNA levels of two markers of fibrosis (α-SMA and Col1α1) were significantly increased in HSCs from both transwell systems, while there was no significant change in Col3α1 mRNA levels (Fig 1H–1O). These data suggest that sja-let-7 of *S*. *japonicum* worms is involved in activation of HSCs.

### *Sj*EVs transfer sja-let-7 into HSCs and reduced their activation

**Fig 2:**
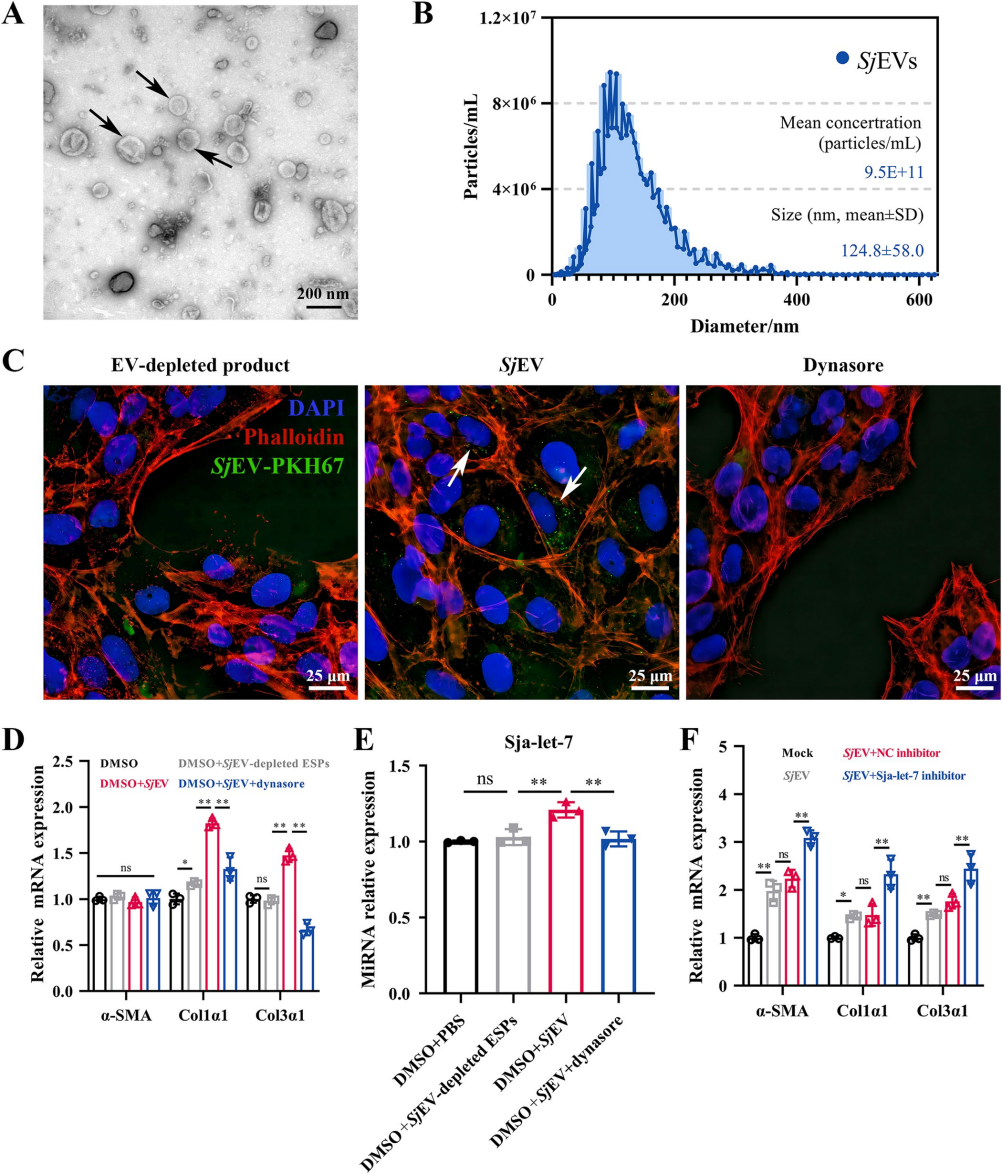
*S*. *japonicum* worm-derived EVs transfer sja-let-7 into HSCs and reduced their activation. (A) TEM analysis for *Sj*EVs. Black arrows indicate the cup-shape EVs. Scale bar, 200 nm. (B) NTA analysis for *Sj*EVs. (C) Microscopy image of PKH67-labelled *Sj*EVs (green) incubated with LX-2 cells for 2 h. White arrows indicate *Sj*EVs labelled with PKH67. Scale bar, 25 μm. (D) Detection of α-SMA, Col1α1 and Col3α1 mRNA expression of the LX-2 cells (n = 3). (E) Detection of sja-let-7 in the LX-2 cells (n = 3). (F) Detection of α-SMA, Col1α1 and Col3α1 mRNA expression of the LX-2 cells (n = 3). All graph data are expressed as the mean ± SD of at least three biological replicates per group. *P< 0.05, **P< 0.01, ns, not significant. Abbreviation: *Sj*: *S*. *japonicum*; TEM: transmission electron microscope; NTA: nanoparticle tracking analyses; NC: negative control.

A previous transwell study reported that *S*.*mansoni* worms influenced the functions of T helper (Th) cells in the lower layer via EVs [19]. The involvement of *Sj*EVs in activation of HSCs was further verified using EVs derived from *S*. *japonicum* worms. The EVs were characterized based on the vesicle size and protein content. As shown in Fig 2A, enrichment of typical EVs with a typical cup-like morphology was confirmed by transmission electron microscopy (TEM). Nanoparticle tracking analysis (NTA) revealed that the mean sizes of *Sj*EVs were 124.8±58.0 nm and original concentration of *Sj*EVs were 9.5×10^11^/ mL (Fig 2B). Additionally, the presence of schistosome EV-associated proteins (actin, tubulin, HSP60, HSP90, GAPDH, CD63, and annexin) [18] was verified by liquid chromatography/mass spectrometry (S2 and S3 Tables).

Next, the potential involvement of *Sj*EVs on activation of HSCs was investigated using purified *Sj*EVs labeled with a green fluorescent dye (PKH67) and EV-depleted ESPs (Excretory-secretory products), which was confirmed by showing no particles under TEM, was taken as a control (S2A Fig). Following treatment with PKH67-labeled EVs for 2 h, uptake of *Sj*EVs by HSCs cells was verified by fluorescence microscopy. The green fluorescence of PKH67-labeled *Sj*EVs was observed in LX-2 cells, demonstrating uptake of *Sj*EVs by HSCs (Fig 2C). This phenomenon could be blocked by the endocytosis inhibitors, dynasore (Fig 2C). Subsequent *in vitro* studies were conducted with *Sj*EVs at a particle concentration of 4.3×10^10^/ mL. Further qPCR analysis revealed that *Sj*EVs treatment significantly increased mRNA levels of two markers of fibrosis (Col1α1, and Col3α1) (Fig 2D). Besides, the relative expression of sja-let-7 and other five *Sj*-miRNAs (sja-bantam, sja-miR-2162, sja-miR-10, sja-miR190 and sja-miR-125b) in HSCs was also up-regulated after *Sj*EVs coculture (Figs 2E and S2B). In addition, the reduction of sja-let-7 abundance significantly increased the mRNA levels of all three markers of fibrosis as compared to the NC inhibitor group (Figs S2C and S2F). Collectively, these results indicate that EVs derived from *S*. *japonicum* worms were involved in general activation of HSC with reduced fibrotic functions in the presences of EV-derived sja-let-7.

### Sja-let-7 reduces the activation of HSCs by targeting the Col1α2/TGF-β/Smad axis *in vitro*

**Fig 3:**
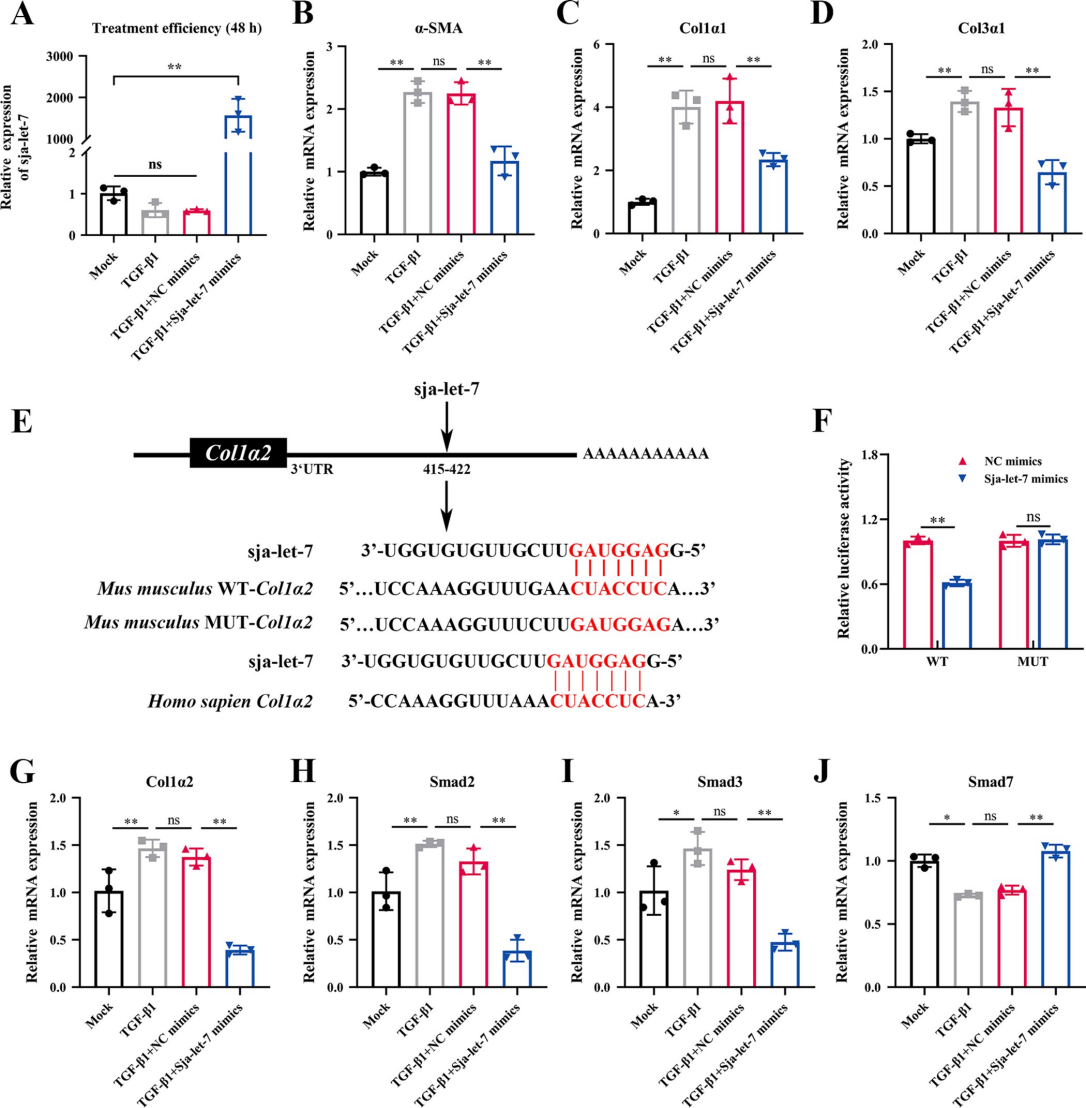
Sja-let-7 reduces the activation of HSCs through Col1α2/TGF-β/Smad axis *in vivo*. (A-D) Treatment efficiency analysis and detection of α-SMA, Col1α1 and Col3α1 mRNA expression towards LX-2 cells after treated with NC or sja-let-7 mimics for 48 h (n = 3). (E) The binding site of sja-let-7 on the 3′-UTR of *M*. *musculus* and *Homo sapien* Col1α2. (F) Results of the dual-luciferase reporter assay (n = 3). (G-J) Detection of Col1α2, Smad2, Smad3 and Smad7 mRNA expression of the LX-2 cells after treated with NC or sja-let-7 mimics (n = 3). All graph data are expressed as the mean ± SD of at least three biological replicates per group. *P< 0.05, **P< 0.01, ns, not significant. Abbreviation: NC: negative control; UTR: untranslated region; WT: wild type; MUT: mutant.

Transforming growth factor-β (TGF-β) is generally considered a potent activator of HSCs [20]. To further clarify the potential impact of sja-let-7 on activation of HSCs, the inhibitory effects of sja-let-7 or NC mimics on TGF-β1-induced activation of HSCs (LX-2 cells) was investigated. After 48 h, the uptake efficiency was assessed by qPCR analysis. The results showed that the mRNA levels of all three markers of fibrosis (α-SMA, Col1α1, and Col3α1) were significantly decreased as compared to the NC group (Fig 3A–3D).

Next, target genes controlled by sja-let-7 were identified to clarify the mechanisms underlying sja-let-7-induced activation of HSCs. Two databases (miRanda [21] and RNAhybrid [22]) were used to identify potential sja-let-7 targets. A Venn diagram illustrated overlap of 1167 potential target genes between the two databases. Therefore, these genes were selected for gene ontology (GO) analysis (S3A Fig and S4 Table). Col1α2, an important component of type I collagen, was linked to the GO terms “collagen trimer (n = 2)”, “extracellular matrix (n = 14)”, and “extracellular matrix part (n = 8)” (S3B Fig and S5 Table). Since let-7 is relatively conserved and there are multiple let-7 family members in the host, to avoid interference of identical seed sequences of let-7 on the screening process, the target genes of all let-7 family members were predicted and cross-referenced with the 1167 target genes of sja-let-7. The results found that Col1α2, which was not identified as a target gene of any let-7 family member, was the only target gene of sja-let-7 (S3C and S3D Fig, S6 and S7 Tables).

Based on the binding site of sja-let-7 on the 3′-untranslated region (UTR) of *Mus musculus* Col1α2, which is homologue to *Homo sapiens* Col1α2, a luciferase reporter plasmid was generated containing the 3′-UTR of *M*. *musculus* Col1α2 flanking the putative sja-let-7 binding sites (Fig 3E). The dual-luciferase reporter assay revealed that as compared to NC mimic-treated cells, sja-let-7 significantly reduced luciferase activity of the Col1α2 construct (Fig 3F). In addition, TGF-β1-mediated upregulation of Col1α2 in HSCs was downregulated by treatment with sja-let-7 mimics (Fig 3G). Furthermore, treatment with a sja-let-7 inhibitor in both transwell systems or *in vitro* treatment with *Sj*EVs (described above) significantly upregulated expression of Col1α2 (S3E–S3J Fig). These results suggest that sja-let-7 inhibited Col1α2 expression via the 3′-UTR.

GO analysis was conducted to further characterize the role of Col1α2. The GO terms associated with to Col1α2 included “transforming growth factor beta receptor signaling pathway”, “response to transforming growth factor beta”, “cellular response to transforming growth factor beta stimulus” and “SMAD binding” (S8 Table). These findings suggest that the Col1α2/TGF-β/Smad axis regulates sja-let-7-induced suppression of HSC activation. To test this hypothesis, components of the TGF-β/Smad signaling pathways were targeted in further experiments. The results of qPCR analysis found that the expression levels of Smad2 and Smad3, which are key components of the TGF-β/Smad signaling pathway, were significantly reduced after treatment with sja-let-7 mimics, while expression of the antagonist Smad7 was increased (Fig 3H–3J). Collectively, these results suggest that sja-let-7 reduces activation of HSCs by targeting the Col1α2/TGF-β/Smad axis.

### Establishment of a BALB/c mouse model of schistosome-induced liver fibrosis

Pathological changes vary among the naturally permissive hosts of *S*. *japonicum*. Therefore, the stages of schistosome-induced liver fibrosis in mice were investigated as a foundation for subsequent analysis. Mice were percutaneously infected with 20 ± 2 cercariae and sacrificed at 2, 4, 6, 8, 10, and 12 weeks post infection (wpi). From 2 to 4 wpi, there were no significant differences in the liver and spleen indices, hematological index, or hydroxyproline content in the liver tissues of mice in the uninfected group, but all indices increased significantly from 6 wpi (S4A–S4F Fig). Histological analysis found no eggs in the liver tissues at 2 wpi. In contrast, by 4 wpi, after maturation and mating of the schistosomes, eggs were found in the liver tissues, although no granulomas were present (S5A Fig). From 6 wpi, egg granulomas were observed by Masson and Sirius red staining (S5A Fig). The extent of granulomas peaked at 8 wpi and then gradually decreased at 10–12 wpi (S5A Fig). To further evaluate the extent of liver fibrosis, the expression profiles of three markers of fibrosis (α-SMA, Col1α1, and Col3α1) were detected by qPCR analysis. The results showed that from 2 to 4 wpi, there was no significant change to the expression levels of the three markers as compared to the uninfected group, while at 6 wpi, the expression levels of all three markers were significantly increased, but then decreased to different degrees at 8–12 wpi (S5B–S5D Fig). Besides, the expression levels of sja-let-7 and its target gene Col1α2 were also detected. Similar to the above indices, the expression levels of both sja-let-7 and Col1α2 started to increase at 6 wpi (S5E–S5F Fig). These results indicate that 6 wpi is the initiation phase of liver fibrosis in BALB/c mice infected with 20 ± 2 cercariae and that the functionality of sja-let-7 may be initiated at this time point.

Analysis of transcriptomic data retrieved from the Gene Expression Omnibus (GEO) database showed that Col1α2 was significantly upregulated in the liver tissues of C57BL/6J mice at the liver fibrosis stage (6 wpi and 7 wpi), suggesting that Col1α2 is a target gene of sja-let-7 and plays a pivotal role in liver fibrosis (S6 Fig).

**Fig 4:**
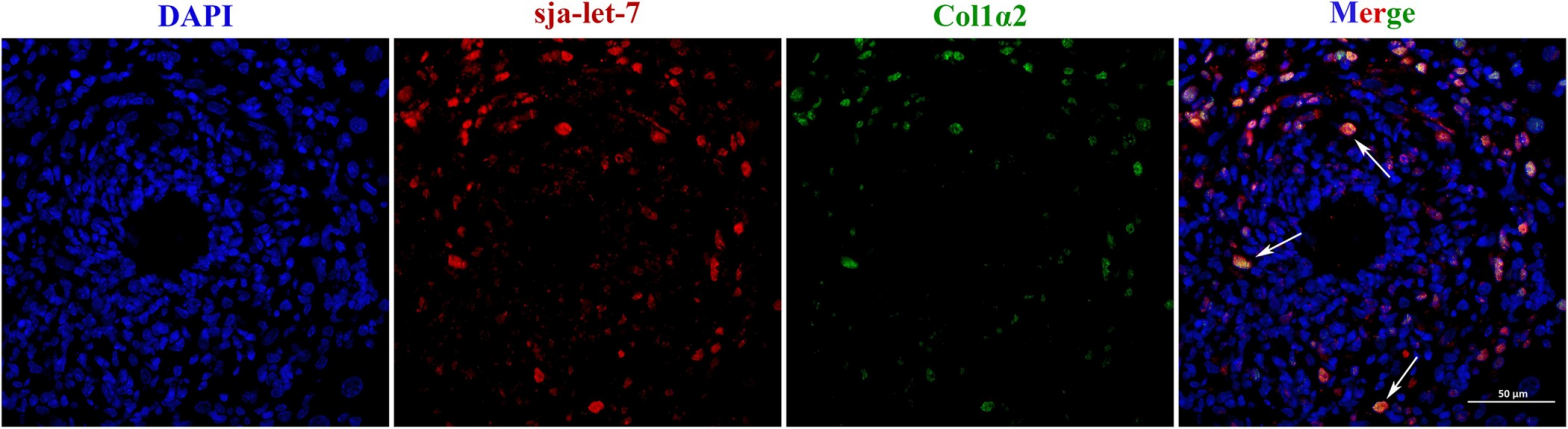
FISH analysis on the liver section. White arrows indicate the cells that co-located with sja-let-7 and Col1α2. Scale bar, 50 μm. FISH: fluorescence *in situ* hybridization.

To further verify the correlation between sja-let-7 and its target gene Col1α2 *in vivo*, fluorescence *in situ* hybridization (FISH) of mouse liver slices was conducted at 6 wpi. As shown in Fig 4, sja-let-7 and Col1α2 were widely expressed around the granuloma and co-localized with the nuclei, thereby confirming a targeting relationship.

Collectively, these results suggest that 6 wpi is the starting point of schistosome-induced liver fibrosis of the BALB/c mice model in the present study and sja-let-7 and its target gene Col1α2 are closely involved in this process.

### Sja-let-7 suppresses schistosome-induced liver fibrosis *in vivo*

**Fig 5:**
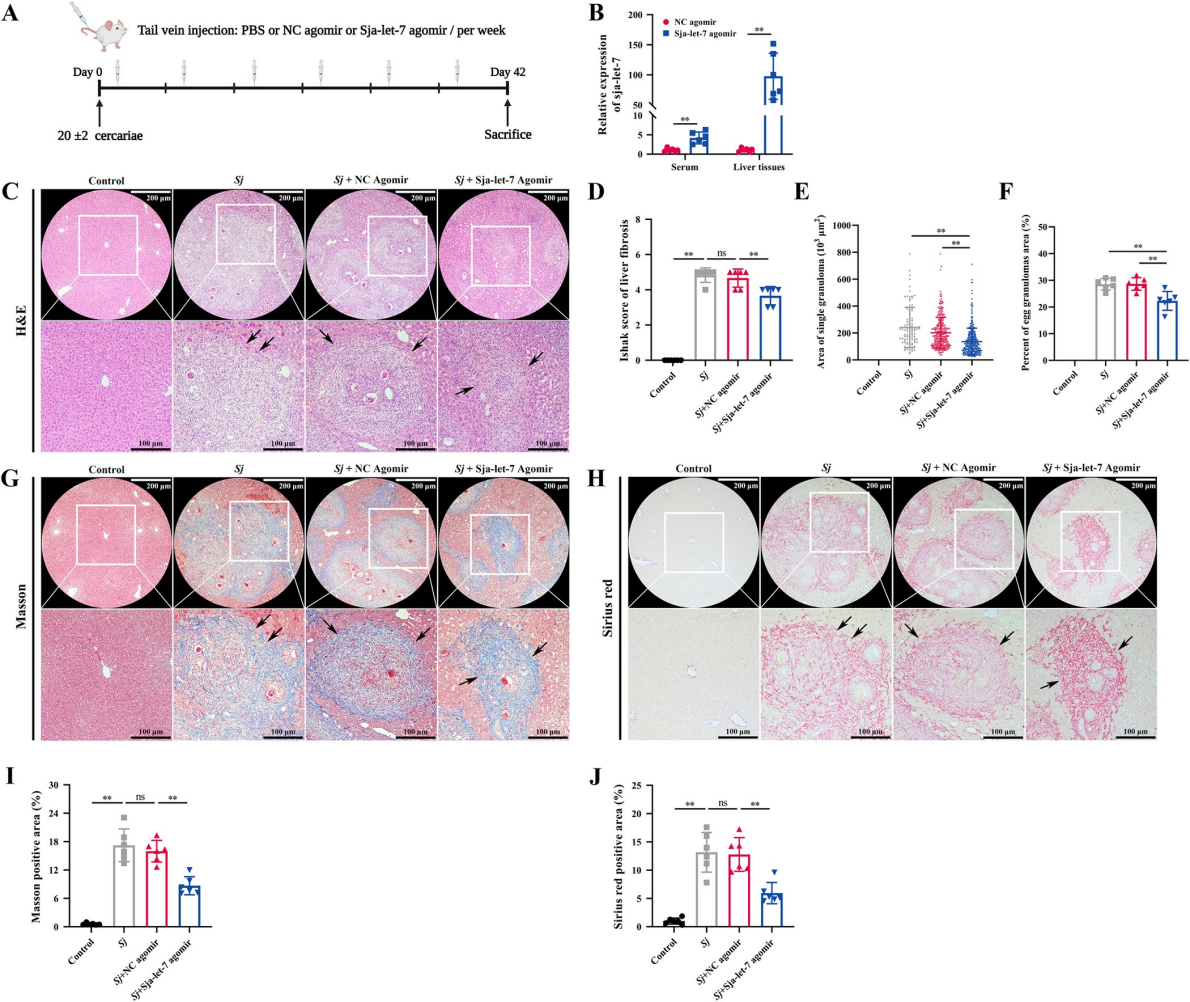
Sja-let-7 attenuates the fibrotic progression of schistosome-induced liver fibrosis. (A) Establishment timeline of schistosome-induced liver fibrosis in a mouse model. (B) Detection of sja-let-7 expression in the liver and serum (n = 6). (C) Liver histological analysis with H&E staining. Black arrows indicate the egg granuloma. Scale bar, 200 μm. *Insets* show a higher magnification of the outlined area. Scale bar, 100 μm. (D) Ishak score of liver fibrosis (n = 6). (E) Area of a single granuloma. (F) Percent of egg granuloma areas (n = 6). (G-H) Liver histological analysis with Masson and Sirius red staining. Black arrows indicate the positive staining area. Scale bar, 200 μm. *Insets* show a higher magnification of the outlined area. Scale bar, 100 μm. (I-J) Positive area of Masson and Sirius red staining (n = 6). All graph data are expressed as the mean ± SD of at least three biological replicates per group. *P< 0.05, **P< 0.01, ns, not significant. Abbreviation: *Sj*: *S*. *japonicum*; NC: negative control; H&E: hematoxylin and eosin.

As mentioned above, 6 wpi is the starting point of schistosome-induced liver fibrosis in BALB/c mice. Therefore, in this experiment, mice were infected with *S*. *japonicum* and administered sja-let-7 agomir, experimentally mimicing the endogenous parasite miRNA function, through the tail vein once per week for six weeks (Fig 5A). Analysis of serum and liver tissues from mice injected with sja-let-7 agomir for 6 weeks showed that the miRNAs in both samples were significantly upregulated (Fig 5B), indicating that sja-let-7 is processed and matured in mice. Administration of sja-let-7 agomir markedly ameliorated hepatosplenomegaly, as indicated by the reductions in the liver (aspartate transaminase (AST), alanine transaminase (ALT), and hydroxyproline), spleen, and hematological indices (S7A–S7J Fig). Besides, hematoxylin and eosin (H&E) staining of liver sections showed that the Ishak score of liver fibrosis and areas with a single egg granuloma were significantly reduced in the sja-let-7 agomir group as compared to the control group (Fig 5C–5F). Furthermore, Masson and Sirius red staining of liver sections showed significantly reduced collagen deposition in the sja-let-7 agomir-treated mice (Fig 5G–5J).

**Fig 6:**
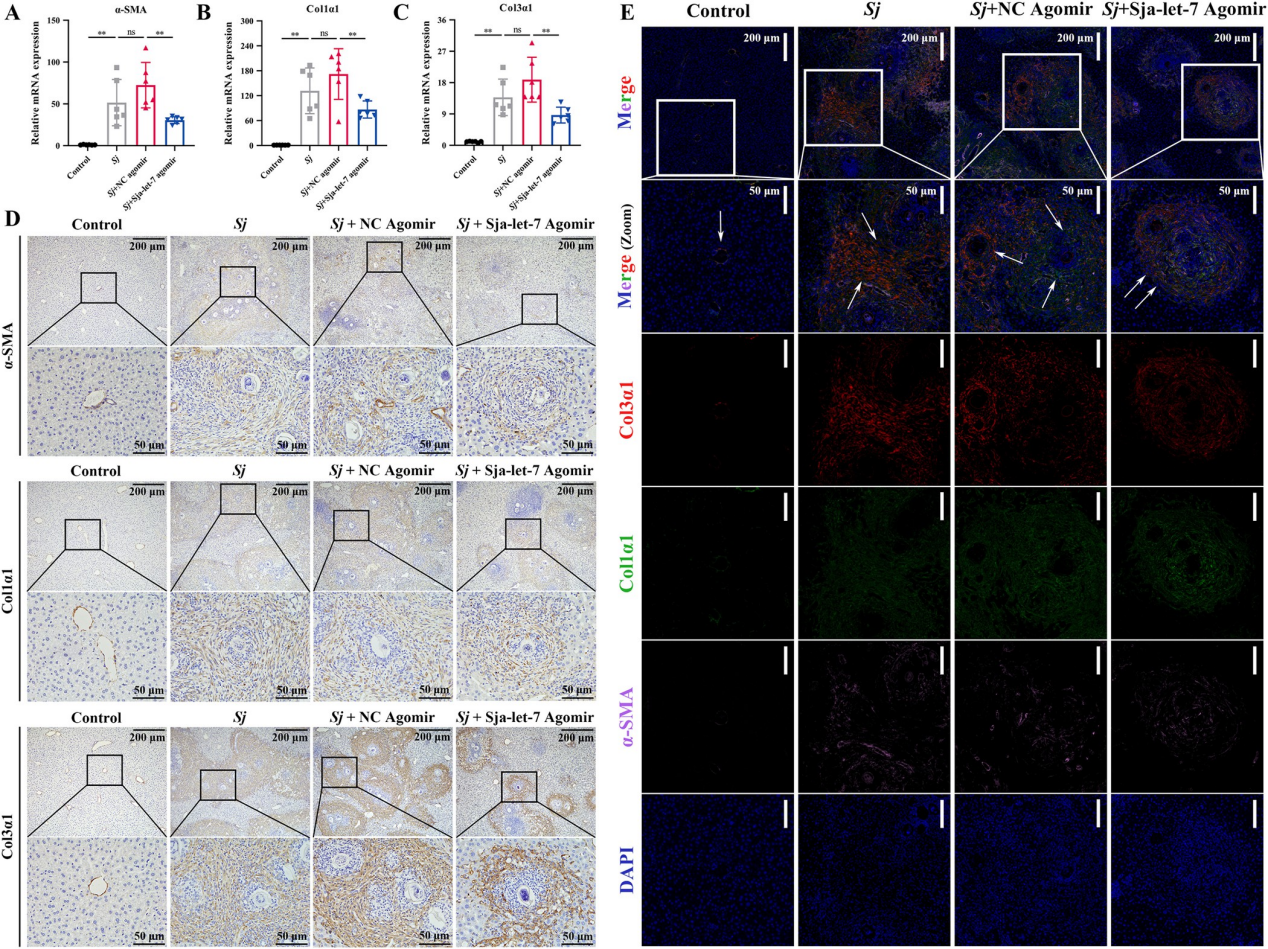
Sja-let-7 reduced the expression of fibrotic markers after treated with sja-let-7 agomir. (A-C) Detection of α-SMA, Col1α1 and Col1α3 mRNA expression in the liver (n = 6). (D) Liver IHC analysis of α-SMA, Col1α1 and Col1α3. Scale bar, 200 μm. *Insets* show a higher magnification of the outlined area. Scale bar, 50 μm. (E) Immunofluorescence analysis of α-SMA, Col1α1 and Col1α3 after treated with sja-let-7 agomir. White arrows indicate the egg granuloma. Scale bar, 100 μm. *Insets* show a higher magnification of the outlined area. Scale bar, 50 μm. All graph data are expressed as the mean ± SD of at least three biological replicates per group. *P< 0.05, **P< 0.01, ns, not significant. Abbreviation: *Sj*: *S*. *japonicum*; NC: negative control; IHC: immunohistochemical analysis.

To determine the role of sja-let-7 on liver fibrosis, qPCR analysis was conducted of the liver tissues of mice from all groups. The results showed that the mRNA levels of all three markers of fibrosis (α-SMA, Col1α1, and Col3α1) were significantly reduced (Fig 6A–6C). Next, both the detection of the 3 fibrotic markers separately using immunohistochemistry (IHC) and the combined observation of the 3 fibrotic markers using immunofluorescence showed a significant decrease of positive area in the sja-let-7 agomir group compared to the infected group (Figs 6D–6E and S7K–S7P). In addition, enzyme linked immunosorbent assay (ELISA) revealed that the serum levels of α-SMA were also significantly reduced in the sja-let-7 agomir group (S7Q Fig).

The process of liver fibrosis is accompanied by the secretion of large amounts of inflammatory cytokines [23]. So, several inflammatory cytokines (IL-1β, IL-6, TNF-α, and HMGB1) were selected for qPCR analysis. The results revealed that the expression levels of all indices were significantly reduced in the sja-let-7 agomir-treated mice (S7R–S7U Fig).

Taken together, these findings indicate that sja-let-7 attenuated progression of schistosome-induced liver fibrosis and decreased liver inflammation *in vivo*.

### Sja-let-7 suppression of schistosome-induced liver fibrosis is mediated by the Col1α2/TGF-β/Smad axis

**Fig 7:**
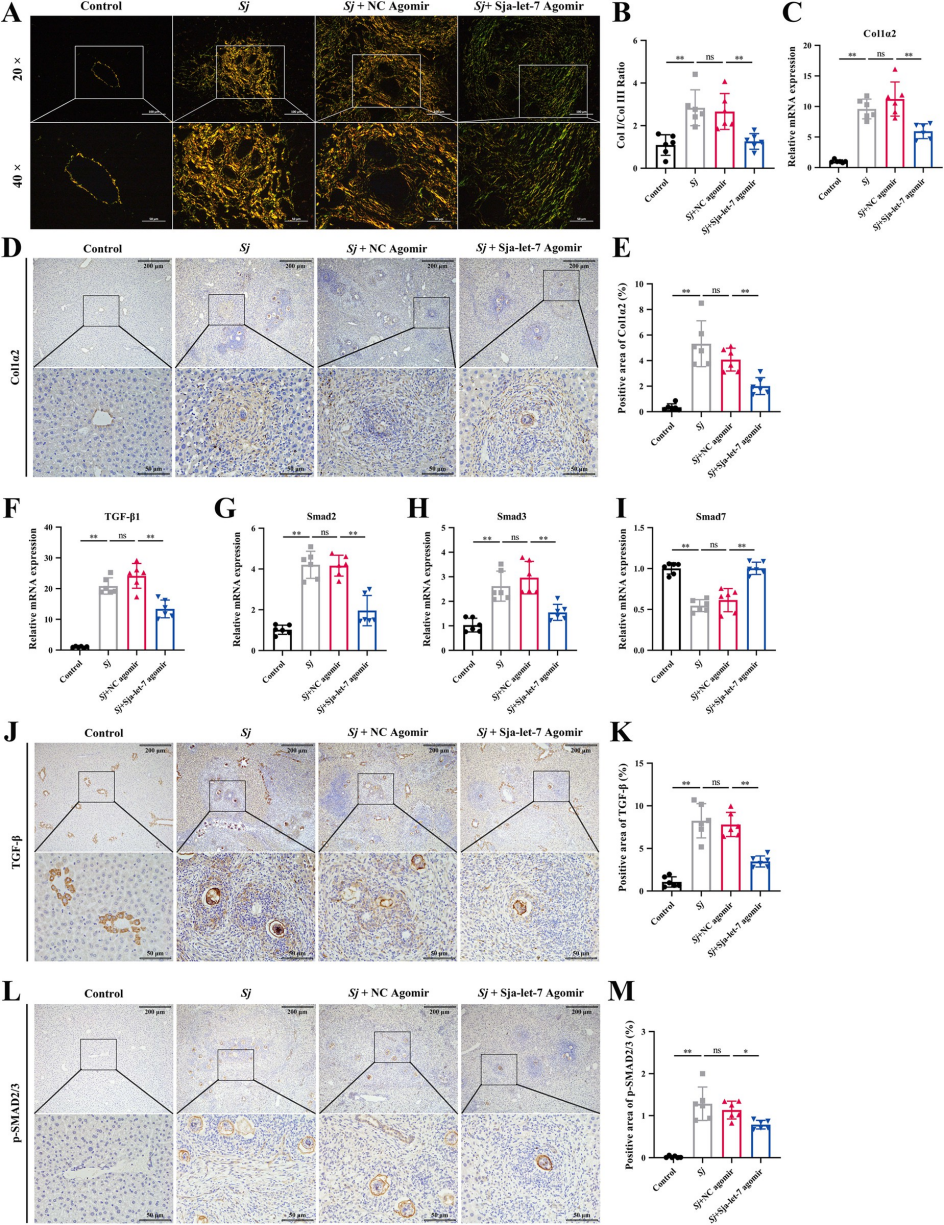
Sja-let-7 suppressed the schistosome-induced liver fibrosis is mediated via Col1α2/TGF-β/Smad axis. (A) Polarization microscopy observation of type I and type III collagen fibers. Scale bar, 100 μm. *Insets* show a higher magnification of the outlined area. Scale bar, 50 μm. (B) Ratio of type I and type III collagen fibers (n = 6). (C) Detection of Col1α2 mRNA expression in the liver (n = 6). (D) Liver IHC analysis of Col1α2. Scale bar, 200 μm. *Insets* show a higher magnification of the outlined area. Scale bar, 50 μm. (E) Positive area of Col1α2 (n = 6). (F-I) Detection of TGF-β, Smad2, Smad3 and Smad7 mRNA expression in the liver (n = 6). (J) Liver IHC analysis of TGF-β. Scale bar, 200 μm. *Insets* show a higher magnification of the outlined area. Scale bar, 50 μm. (K) Positive area of TGF-β (n = 6). (L) Liver IHC analysis of p-smad2/3. (M) Positive area of p-smad2/3 (n = 6). All graph data are expressed as the mean ± SD of at least three biological replicates per group. *P< 0.05, **P< 0.01, ns, not significant. Abbreviation: *Sj*: *S*. *japonicum*; NC: negative control; Col I: type I collagen fiber; Col III: type III collagen fibers; IHC: immunohistochemical analysis.

**Fig 8:**
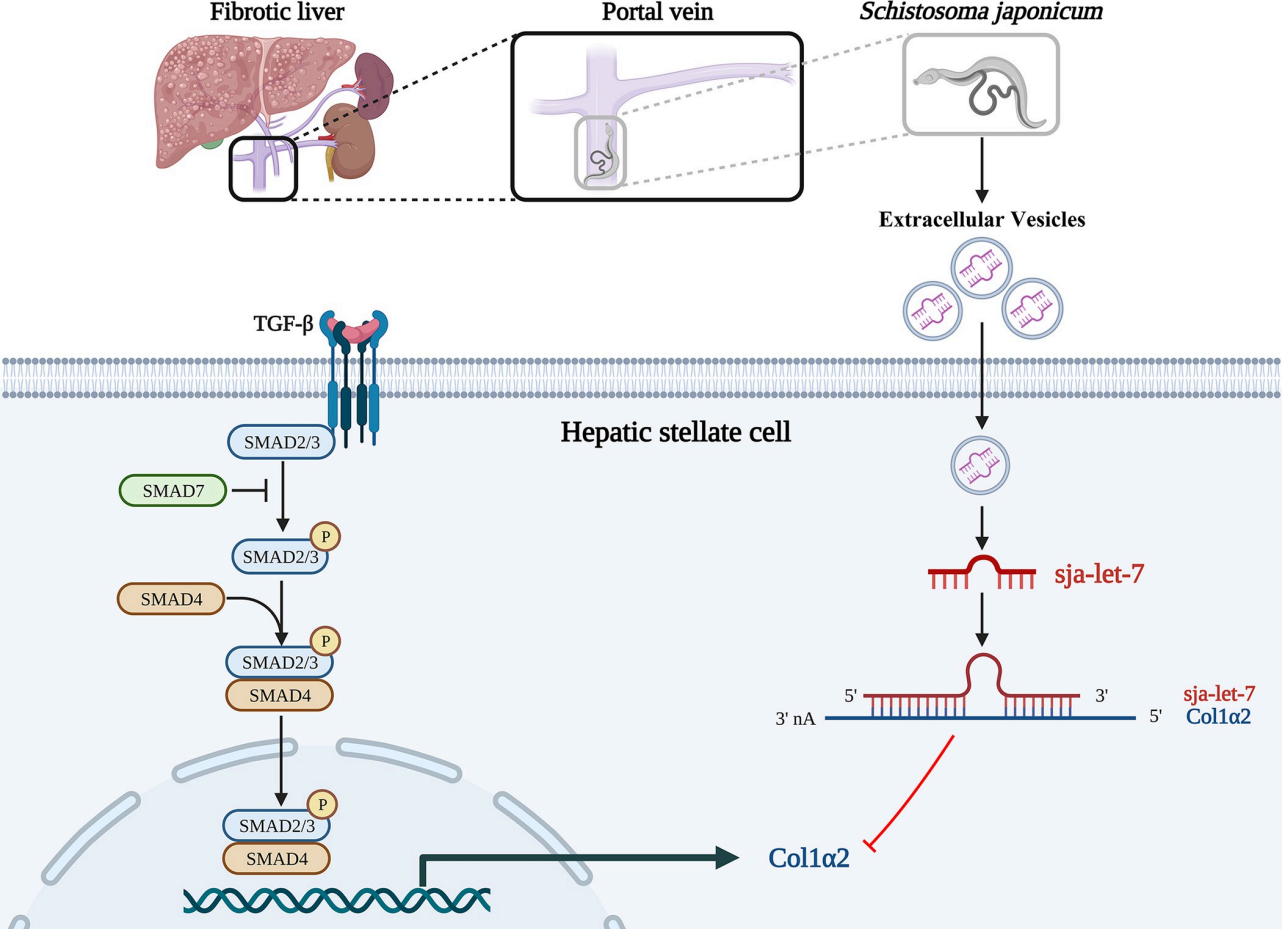
Graphical abstract of the mechanism. The *S*. *japonicum* worms dwelling in the host portal vein release *Sj*EVs that contain sja-let-7 to suppress the schistosome-induced liver fibrosis via Col1α2/TGF-β/Smad axis. This figure was created with Biorender.com.

To determine whether sja-let-7 also regulates liver fibrosis via the Col1α2/TGF-β/Smad axis *in vivo*, the expression levels of Col1α2 and components of the TGF-β/Smad signaling pathway were determined. First, the contents of type I and type III collagen fibers were quantified by polarization microscopy. As shown in Fig 7A and 7B, the content of type I collagen fibers, as indicated by bright red or yellow staining, was significantly reduced in the liver tissues of mice in the sja-let-7 agomir group. As an important component of type I collagen, the expression level of Col1α2 was measured by qPCR and immunohistochemical analyses. The results revealed that Col1α2 expression was significantly reduced at both the mRNA and protein levels, indicating that uptake of sja-let-7 miRNA agomir inhibits the sja-let-7 target gene, Col1α2 (Fig 7C–8E).

Next, sja-let-7-induced suppression of liver fibrosis through the TGF-β/Smad signaling pathway was verified. Notably, sja-let-7 agomir treatment significantly reduced the expression levels of TGF-β1, Smad2, Smad3, and p-Smad2/3, while on the TGFβ-antagonist Smad7 there was an opposite effect (Fig 7F–7M).

Collectively, these results confirm that sja-let-7-induced suppression of schistosome-induced liver fibrosis is mediated via the Col1α2/TGF-β/Smad axis.

## Discussion

The present study provides evidence of a functional role of sja-let-7 in *S*. *japonicum* worm-derived EVs in schistosome-induced liver fibrosis. Comparisons of *Sj*-miRNAs from *S*. *japonicum* worm- and egg-derived EVs and primary HSCs of infected mice found that in addition to the eggs, which are the central part of a granuloma, the worm may also deliver informative substances through EVs, such as sja-let-7, to regulate activation of the host HSCs as a form of long-term symbiosis and the anti-fibrotic effect of sja-let-7 is mediated via the Col1α2/TGF-β/Smad axis (Fig 8).

Accumulating evidence shows that *Sj*-miRNAs in the schistosome EVs play pivotal roles in the host-parasite interaction [24]. Liu et al. [15] reported that EVs released by paired adult *S*. *japonicum* worms are primarily absorbed by mouse peripheral blood monocytes, resulting in increased production of TNF-α, which is essential for the survival and egg production of *S*. *japonicum*. Overexpression of sja-miR-125b and sja-bantam has been implicated as the main contributor to this phenomenon. With the use of a transwell system with Th cells in the lower well, Meningher et al. [19] showed that adult *S*. *mansoni* worms in the upper well differentially restricted Th2 polarization by interfering with Th2-specific transcription, which was partially mediated by sma-miR-10 in the *S*. *mansoni* EVs. Another study found that activation of the NF-kappa B target gene Map3k7, which is a critical transcription factor for Th2 differentiation, was inhibited by sma-miR-10 [25]. In the present study, a similar transwell system was established with adult *S*. *japonicum* worms in the upper wells and HSCs in the lower wells to verify the hypothesis that substances carried by EVs and other soluble secreted factors of adult schistosome worms can directly regulate activation of HSCs. The presence of four *Sj*-miRNAs in both primary HSCs of infected mice and *S*. *japonicum* worm-derived EVs was confirmed. The use of recent methodologies to effectively isolate schistosome EVs further revealed that EVs derived from *S*. *japonicum* worms could be absorbed by HSCs, indicating the miRNA-loaded schistosomal EVs could transfer from the upper well to the lower well of the transwell system [26,27]. In addition, a transwell system with adult mated male worms in the upper wells confirmed the presence of *Sj*-miRNAs from MM-derived EVs in HSCs [18]. Although the function of these *Sj*-miRNAs in activation of HSCs remains unclear, the results of this study provide evidence that single-sex or bisexual infected male and female schistosome worms have different effects on liver fibrosis [28–30].

Among the four *Sj*-miRNAs from both primary HSCs of infected mice and *S*. *japonicum* worm-derived EVs, sja-let-7 was selected for further study, which has not shown high expression in schistosome egg-derived EVs based on current available studies [12,14,16]. Members of the miRNA let-7 family, which were originally discovered in *Caenorhabditis elegans*, control the timing of stem-cell division and differentiation of nematodes [31]. Other biological functions of let-7 family members include neuromusculature development and adult behaviors in flies [32], limb development in chicken and mice [33], and cell proliferation and differentiation [34]. Other studies have reported the involvement of sja-let-7 in the transformation from a miracidium to sporocyst in the snail as an intermediate host and higher expression in single-sex infected than bisexual female worms, suggesting potential functions in regulation of the sexual status of female worms [35,36]. In mammals, many let-7-family members function as tumor suppressors in a variety of cancers [37,38]. Recent studies have revealed that some let-7-family members act as anti-fibrotic factors in various fibrotic diseases. Let-7a-5p, which is highly expressed in human bone marrow mesenchymal stem cell-derived EVs, was reported to inhibit TGF-β-induced fibroblast activation and collagen secretion *in vitro* and extenuate capsular stiffness *in vivo* by targeting TGFβRI [39]. Let-7b is also reported to inhibit *S*. *japonicum* recombinant P40 protein-induced activation of HSCs by direct targeting of Col1α1 [40]. In another study, transfection with a lentivirus encoding let-7b significantly reduced liver fibrosis in mice infected with *S*. *japonicum* [41]. These findings suggest that some members of the let-7-family have the potential to negatively regulate fibrotic diseases. However, to our best knowledge, there have been no reports of sja-let-7 in schistosome-induced liver fibrosis. Based on the same seed sequence and highly conserved functions across species, sja-let-7 mimics and agomir were synthesized and applied in *in vitro* and *in vivo* to verify the hypothesis that substances carried by EVs of adult schistosome worms can directly regulate activation of HSCs. The results of the present study showed that sja-let-7 significantly inhibited activation of HSCs *in vitro* and attenuated progression of liver fibrosis of mice infected with *S*. *japonicum*. A previous study by our group found that the increase of sja-let-7 by injecting sja-let-7 mimics to the infected mice had no impact on egg burden, indicating the anti-fibrotic effect was not caused by decreased parasite reproduction, but rather the direct activities of sja-let-7 in the liver [42]. In contrast, an inhibitor of sja-let-7 induced stronger activation of HSCs *in vitro*.

To further elucidate the molecular mechanisms of sja-let-7 in liver fibrosis, FISH analysis and a dual-luciferase reporter assay were conducted to identify direct target genes that might be controlled by sja-let-7 rather than other let-7-family members. The results confirmed that Col1α2 is a direct target of sja-let-7. A previous study also confirmed the relationship between let-7 carried by the EVs of *S*. *mansoni* and Col1α2 of the host [43]. Schistosome-induced liver damage resulted in fibroblast activation and extracellular matrix accumulation, especially type I collagen, which is composed of one Col1α2 and two Col1α1 chains [44]. In the present study, the upregulation of sja-let-7 down-regulated Col1α2 expression both *in vitro* and *in vivo*. In addition, upregulation of sja-let-7 inhibited expression of key components of the TGF-β/SMAD signaling pathway, which promotes liver fibrosis by activation of HSCs and production of type I collagen [45,46]. Thus, sja-let-7 suppresses liver fibrosis by inhibiting the Col1α2/TGF-β/Smad axis, as verified by bioinformatics analysis. Furthermore, after treatment of sja-let-7 agomir, the expression of inflammatory cytokines (IL-1β, IL-6, TNF-α, and HMGB1) were also reduced, suggested alleviation of liver inflammation.

Overall, the results of this study demonstrate that sja-let-7 carried by the EVs of *S*. *japonicum* worms reduced activation of HSCs by targeting Col1α2 and further inhibited progression of liver fibrosis by mediating the TGF-β/Smad signaling pathway. These findings expand current understanding of host-parasite interactions during schistosomiasis and identified a promising target for treatment of schistosomiasis.

## Materials and methods

### Ethics statement

All animal experiments were performed in accordance with the guidelines of the Committee for the Care and Use of Laboratory Animals of the Shanghai Veterinary Research Institute, Chinese Academy of Agricultural Sciences (Shanghai, China, permit no. SYXK-20160010). The study protocol was approved by the Ethics and Animal Welfare Committee of the Shanghai Veterinary Research Institute, Chinese Academy of Agricultural Sciences (Shanghai, China, experiment no. SV-20230505-03).

### Laboratory animals, parasites and infection

Specific-pathogen-free (SPF) male BALB/c mice (6–8 weeks old; body weight 18 ± 2 g), KM mice (6–8 weeks old; body weight 20 ± 2 g) and New Zealand rabbits (7–8 weeks old; body weight 2 kg) were purchased from Shanghai Jiesijie Laboratory Animal Co., Ltd. (Shanghai, China) and housed in SPF-grade animal rooms at the Shanghai Veterinary Research Institute, Chinese Academy of Agricultural Sciences (Shanghai, China). Animals were randomly allocated to certain groups before the start of the study.

*S*. *japonicum* cercariae were obtained from the National Reference Laboratory for Animal Schistosomiasis, Shanghai Veterinary Research Institute, Chinese Academy of Agricultural Sciences (Shanghai, China). BALB/c mice, KM mice or New Zealand rabbits were percutaneously infected by applying cercariae (the number of cercariae varies according to the experiments and will be described in the following method sections) to the shaved skin of the abdomen. At a particular point in time, depending on different experiments, animals were euthanized and paired adult worms were collected through hepatic-portal perfusion as previously described [47]. The mated male (MM) worms were carefully separated from the paired worms under a microscope, then collected.

### Cell culture and transfection

Human hepatic stellate cell line LX-2 and human embryonic kidney 293T (HEK293T) cells were obtained from Boster company (Wuhan, China) and cultured in the Dulbecco’s modified Eagle’s medium (DMEM, Corning, USA), supplemented with 10% heat-inactivated fetal bovine serum (FBS, Gibco, USA), 1% penicillin-streptomycin (Thermo Fisher Scientific, USA) in a humidified incubator at 37°C with 5% CO_2_.

For transfection, when cells had reached a density of 1×10^6^ cells/well in the 6-well plate (or 5×10^5^ cells/well in the 12-well plate), they were transfected with 100 pmol/well (or 40 pmol/well in the 12-well plate) Sja-let-7 mimics/inhibitors (GenePharma, China) or corresponding negative control (NC) mimic/inhibitors with Lipofectamine 3000 (Invitrogen, USA) for 48 h according to the manufacturer’s instructions. An additional group was named as Mock group with liposomal transfection reagent and phosphate buffer solution (PBS, Corning, USA) only. The detailed sequences of miRNA mimics/inhibitors are shown in S9 Table.

For stimulation, when cells had reached a density of 1×10^6^ cells/well in the 6-well plate, they were challenged with 2.5 ng/mL/well recombinant TGF-β1 for 12 h followed by correspondent transfection with Sja-let-7 or NC mimic or with PBS only. Or the cells were stimulated with *Sj*EVs at a final particle concentration of 4.3×10^10^/mL for 2 h.

Each group of cells had at least three technical replicates. Depending on different experiments, cells were harvested for qPCR analyses at a particular point in time.

### Transwell system

BALB/c or KM mice were percutaneously infected with approximately 100 cercariae and worms were collected from the infected mice at 28 days post infection (dpi). Parasites were thoroughly and gently washed three times with 20 mL PBS and then maintained in preheated DMEM. In each transwell system of 12-well plate (PET membrane, pore size 0.4 μm) (Corning, USA), LX-2 cells (5×10^5^ cells/well) were placed in the lower well with 1.5 mL DMEM containing 10% FBS and 1% penicillin-streptomycin. 5 pair adult worms or 10 MM worms (28-day) coming from BALB/c or KM mice were transferred from the previous DMEM culture and placed in the upper well with 900 μL fresh DMEM containing 10% FBS and 1% penicillin-streptomycin. A transwell insert with only an unused schistosomal medium was utilized as a control. Each transwell system had at least three technical replicates and were cultured in a humidified incubator at 37°C with 5% CO_2_. The protocols involving transfection in the transwell system are shown in S8 Fig. Cells were harvested for qRT-PCR 48 h after co-incubation was established.

### Isolation and purification of *Sj*EVs

The isolation of *Sj*EVs was performed as previous described with modification [26,27]. Briefly, New Zealand rabbits were percutaneously infected with approximately 3,000 cercariae and worms were collected through hepatic-portal perfusion at 28 dpi. Worms were gently washed 3–5 times with 50 mL PBS and were microscopically examined to ensure the teguments were intact, the dead or fragmentary ones were discarded. The remaining worms were then maintained in preheated RPMI-1640 culture medium (Corning, USA) containing 1% penicillin-streptomycin at 37°C under 5% CO_2_ at a density of ~15 worm pairs / mL for 2 h. After 2 h incubation, the supernatant was collected and fresh culture medium was added for the next collection (the whole collecting procedure could last for 3–4 days until the worms were less active). The pellets in the collected supernatant were discarded by centrifugation at 2,000× g and 14,000× g for 30 min each at 4°C, respectively. Then, the supernatant was collected and dialyzed in PBS for 24 h at 4°C followed by centrifugal ultrafiltration through a 3K Omega membrane (Pall, USA). The supernatant was then filtered using a 0.22 μm syringe filter (Pall, USA) and a total EV isolation kit (Thermo Fisher Scientific, USA) was used according to the manufacturer’s instructions. The EV pellet was resuspended in 200 μL of PBS and then stored at -80°C until further analysis. The supernatant after pelleting the *Sj*EVs was collected and named as “*Sj*EV-depleted ESPs”. The workflow of *Sj*EVs isolation and purification protocols are shown in S9 Fig. The detailed information for *Sj*EVs collection based on “roadmap of EVs from parasitic helminths” [48] is listed in S1 Data.

### Electron microscopy and NTA

*Sj*EVs or *Sj*EV-depleted ESPs suspension was adsorbed onto 200 mesh formvar-coated grids (Agar Scientific, UK) for 2 min at room temperature (25°C). The grids were then stained with 2% phosphotungstic acid (Solarbio, China) for 2 min and examined under a TEM (FEI, Netherlands). The size distributions of EVs were determined by NTA using the ZetaView system (Particle Metrix, Germany).

### Enzymatic digestion of *Sj*EVs protein, mass spectrometry and data analysis

The purified EVs were dissolved in PBS. To reduce the proteins, dithiothreitol was then added at a final concentration of 10 mM followed by incubation at 37°C for 1.5 h. Iodoacetamine was then added at a final concentration 50 mM to alkylate the proteins followed by incubation at room temperature (RT) in the dark for 40 min. Trypsin was then added at a trypsin to protein ration of 1:50 (w/w) for overnight digestion at 37°C after 4-fold dilution in 25mM NH_4_HCO_3_ buffer to achieve a final urea concentration of less than 2M. Trypsin digestion was stopped by adding trifluoroacetic acid to a final concentration of 1%. The peptides of each sample were desalted on C18 cartridges, concentrated by vacuum centrifugation and reconstituted in 40 μL of 0.1% (v/v) formic acid.

For total protein identification, LC-MS/MS, Thermo Scientific, USA) analysis was performed using a Q Exactive mass spectrometer coupled to an Easy nLC system (Thermo Scientific). Trypsin-digested peptides (~5 μg) were trapped and desalted on Zorbax 300SB-C18 peptide traps (Agilent Technologies, USA) and separated on a C18-reversed phase column (0.15 mm × 150 mm, Column Technology, USA). The Easy nLC system (Thermo Scientific, USA) was used to deliver mobile phases A (0.1% formic acid in HPLC-grade water) and B (0.1% Formic acid in 84% acetonitrile) with a linear gradient of 4–50% B (0–50 min), 50–100% B (50–54 min), and then 100% B (54–60 min) at a flow rate of 250 nL/min. The nanoliter liquid phase separation end was directly connected to the mass spectrometer.

To acquire the MS data, a data-dependent top ten method was used, in which the ten most abundant precursor ions were selected for HCD fragmentation. For survey scans (m/z 300–1800), the target value was determined based on predictive Automatic Gain Control at a resolution of 70,000 at m/z 200 and dynamic exclusion duration of 25 s. Resolution for HCD spectra was set to 17,500 at m/z 200. Normalized collision energy was 27 eV and the under fill ratio, which specifies the minimum percentage of the target value likely to be reached at maximum fill time, was defined as 0.1%.

Data interpretation and protein identification were performed with the MS/MS spectra data sets using the MaxQuant software (v 1.5.5.1, Max Planck Institutes, Germany) (http://www.maxquant.org) [49] against the UniProtKB *Schistosoma japonicum* database (download on March 06, 2023). The search parameters were trypsin enzyme, two missed cleavages, fixed modifications of carbamidomethyl, variable modifications of oxidation, a fragment ion mass tolerance of 0.10 Da, and peptide tolerance of 20 ppm. Only proteins with at least two peptides (filtered by an ion score ≥ 20 and false discovery rate of <0.01) uniquely assigned to the respective sequence were considered as identified.

### *Sj*EVs uptake experiment

Isolated *Sj*EVs were labeled with PKH67 using a Green Fluorescent Labeling Kit (Sigma Aldrich, USA), and the procedures were performed according to the manufacturer’s protocol. Briefly, *Sj*EVs at a particle concentration of 4.3×10^10^/mL were stained with PKH67 dye in 500 μL of Diluent C fluid for 5 min at RT. Next, 1 mL 1% bull serum albumin (BSA, Yeasen, China) was added to stop the labeling process. Then, the labeled *Sj*EVs were re-purified via ultracentrifugation at 100,000× g with PBS rinsing for 90 min. As a control for non-specific labeling of cells, *Sj*EV-depleted ESPs was PKH67-stained, washed, and added to the cells as a parallel experiment. The whole procedure was conducted at 4°C. Then, the PKH67-labeled *Sj*EVs were co-incubated with LX-2 for 2 h in a humidified incubator at 37°C with 5% CO_2_. For the inhibitor treatment, the cells were pretreated with 150 μM dynasore (Sigma Aldrich, USA) for 30 min at 37°C and then incubated with PKH67-labeled *Sj*EVs for 2 h as described above in the presence of inhibitors. Afterward, the culture medium was discarded, and then the cells were washed in PBS three times, fixed with 4% formaldehyde solution (Servicebio, China) for 15 min and washed twice more with PBS. After this, cells were then incubated with TRITC phalloidin (Yeasen, China) for 30 min and nuclei were stained with 4′,6-diamidino-2-phenylindole (DAPI, Sigma Aldrich, USA) for 3 min. After washed in PBS three times to remove the remaining DAPI, the cells were observed using a fluorescence microscopy (Olympus, Japan).

### Establishment of *Sj*-induced liver fibrosis model

To determine the initiation time of *Sj*-induced liver fibrosis in BALB/c mice with the infection dose of 20±2 cercariae, the overall course of pathology from 0−12 wpi were observed at first. 21 mice were percutaneously infected with 20±2 cercariae to the shaved skin of the abdomen and sacrificed at 2, 4, 6, 8, 10 and 12 weeks post infection (wpi) (n = 3). Another 3 uninfected mice were placed as the uninfected group. Liver, spleen tissues and blood sample of each mouse were collected for further experiments. The distribution of samples for different experiments is shown in S10 Fig.

In the subsequent *in vitro* RNAi experiments, 18 BALB/c mice which were percutaneously infected with 20±2 cercariae for establishment of liver fibrosis mouse model were divided into *Sj*, *Sj*+NC agomir, *Sj*+Sja-let-7 agomir groups (n = 6). In the *Sj*+NC agomir and *Sj*+Sja-let-7 agomir groups, mice were injected with 120 μL of 1 OD (optical density) NC or Sja-let-7 agomir (GenePharma, China) via the tail vein once a week for 6 weeks, respectively. In another normal saline groups (control) group, mice were uninfected with cercariae and were injected with 120 μL PBS via the tail vein once a week for 6 weeks (n = 6). All mice were sacrificed at 6 wpi, liver, spleen tissues and blood sample of each mouse were collected for further experiments S10 Fig. The workflow of *in vitro* RNAi experiments of *Sj*-induced liver fibrosis model is shown in Fig 5A. The detailed sequences of miRNA agomirs are shown in S9 Table.

### Liver and spleen index

Whole spleens and livers were collected and the size was measured. Then the extent of liver damage was assessed macroscopically including the changes in liver color, stiffness and the prevalence of nodules. The livers and spleens were weighed and the liver and spleen indexes were expressed as ratio of the respective organ to body weight [50].

### Hematological analyses

Blood samples (~150 μL/mouse) were collected into K2 EDTA tubes (Solarbio, China), and complete blood count (CBC) assay was conducted on Mindray BC-6800 Plus analyser (Mindary, China). White blood cells were divided into five categories: neutrophil (Neu), lymphocyte (Lym), monocyte (Mon), eosinophil (Eos) and basophil (Bas).

### Liver enzyme quantification

For assessment of mouse liver function, the hydroxyproline, aminotransferase (ALT) and aspartate aminotransferase (AST) of the liver tissues were measured by commercial kits (Nanjing Jiancheng Bioengineering Institute, China) according to the manufacturer’s instructions.

### ELISA

Blood samples (~250 μL/mouse) were collected and was allowed to stand for 10 min followed by centrifugation at 3,000× g for 15 min at 4°C. The serum in the supernatant was used to detect the α-SMA levels through a commercial mouse α-SMA ELISA kit (MLBio, China). The procedures were performed according to the manufacturer’s instructions.

### Histological, IHC and immunofluorescence analysis

The liver samples were fixed with 4% formaldehyde solution at RT for 48 h, dehydrated in ethanol, cleared in xylene, embedded in paraffin, and sliced into 5 μm sections for pathological observation using standard H&E staining (Servicebio, China). The extent of liver fibrosis was determined using Masson’s trichrome staining (Servicebio, China) and Sirius red staining (Servicebio, China). In H&E staining slices, liver fibrosis and inflammation were assessed through the Ishak index score, which is a scoring system commonly used in liver fibrotic disease [51]. The area of single egg granuloma and total granuloma area were measured using ImageJ software (National Institutes of Health, USA). In Masson and Sirius red staining slices, the percentage of positive staining area was also measured using ImageJ software. Besides, polarization microscopy (ZEISS, USA) was used to differentiate type I and type III collagen fibers and to quantitate their proportions in the Sirius red staining slices [52,53]. Type I collagen fibers were recognized by its thick fibers and bright red or yellow staining, while type III collagen fibers were characterized by slander fibers and green staining. The quantitative measurements of type I and type III collagen fibers were measured using ImageJ software.

IHC was performed using serial paraffin sections as above, which were incubated with primary antibodies of α-SMA, Col1α1, Col1α2, Col3α1, TGF-β or p-SMAD2/3 overnight at 4°C, and the sections were then incubated with the indicated secondary antibodies. The percentage of stained positive areas was quantified by ImageJ software.

In order to further observe the degree of liver fibrosis, immunofluorescence experiments were performed as previously described with some modifications [14]. Briefly, the liver paraffin sections were dewaxed and rehydrated with gradient xylene/alcohol and PBS. Cover objective tissues with 5% BSA at RT for 30 min and slides were incubated with the first primary antibody overnight at 4°C, after washed three times with PBS, slides were incubated with secondary antibody at RT for 50 min in dark condition. The procedure was repeated twice with the second and the third set of primary and secondary antibodies. In order to remove the primary antibodies and secondary antibodies combined with tissue, microwave treatments were conducted between each set of antibodies. Afterward, nuclei were stained DAPI for 10 min. After washed in PBS three times to remove the remaining DAPI, the slides were observed using a fluorescence microscopy (Olympus, Japan). The percentage of positive areas was quantified by ImageJ software.

The detailed information of antibodies used in IHC and immunofluorescence experiment is presented in S10 Table.

### Bioinformatics analysis of miRNA targets

Two bioinformatic analysis software, miRanda [21] and RNAhybrid [22] were applied to predict mRNA targets of sja-let-7 in *Mus musculus*. The mRNAs were based off the latest dataset of *M*. *musculus* whole transcriptome (GenBank accession no. PRJNA20689). The miRNA potential targeted genes predicted by both software were selected for the following gene ontology (GO) analysis [54] and Kyoto Encyclopedia of Genes (KEGG) analysis [55]. Besides, target genes of miRNAs from the *M*. *musculus* let-7 family (mmu-let-7a-5p, mmu-let-7b-5p, mmu-let-7c-5p, mmu-let-7d-5p, mmu-let-7e-5p, mmu-let-7f-5p, mmu-let-7g-5p, mmu-let-7i-5p, mmu-let-7j, mmu-let-7k and mmu-miR-98-5p) were also predicted and the overlapping targets among them were cross-referenced with the predict targets of sja-let-7 in *M*. *musculus*. Afterwards, Col1α2 (GenBank accession no. NM_007743.3) was selected for further verification.

### Dual-luciferase reporter assay

To confirm Col1α2 was a target of sja-let-7, wild-type or mutant 3’UTRs of Col1α2 were chemically synthesized (GenePharma, China) and then cloned into the pmirGLO luciferase plasmid (Promega, USA). The HEK293T cells were seeded in a 24-well plate (3 × 10^5^ cells/well). When the cells density reached up to 70%, 25 pmol sja-let-7 or NC mimics, together with 500 ng wild-type Col1α2 3’UTR plasmid or mutant Col1α2 3’UTR plasmid, were transfected into the HEK293T cells using Lipofectamine 3000. Subsequently, the cells were cultured for 48 h and then collected. A Dual-luciferase Reporter Assay Kit (Promega, USA) was used to detect the effect of sja-let-7 on the luciferase activity of the Col1α2 3’UTR plasmid.

### FISH

To demonstrate the relationship between Col1α2 and sja-let-7 on liver sections, FISH was performed. Briefly, according to the tissue fixation time, the slices are boiled in the retrieval solution for 10–15 min and naturally cooled. Add 20 μg/mL proteinase K (Servicebio, China) working solution to cover objectives and incubate at 37°C for 15 min. Wash in pure water, then wash three times in PBS on a rocker device, 5 min each. Pre-hybridization solution was added to each section and incubate for 1 h at 37°C. Then, remove the pre-hybridization solution, add the sja-let-7 probe hybridization solution with concentration of 500nM, and incubate the section in a humidity chamber and hybridize overnight at 40°C. Then, remove the hybridization solution. Wash sections in 2×SSC (Servicebio, China) for 10 min at 37°C, in 1×SSC two times for 5 min each at 37°C, and wash in 0.5×SSC for 10 min at RT. Discard the solution, add the Col1α2 probe hybridization solution with concentration of 500nM, and incubate the section in a humidity chamber and hybridize overnight at 40°C. Remove the hybridization solution. Sections were washed with 2×SSC, 1×SSC, 0.5×SSC for 5 min each at 37°C, respectively. Afterward, nuclei were stained DAPI for 10 min. The slides were observed using a fluorescence microscopy (Olympus, Japan).

The detailed information of probes used in FISH experiment is presented in S11 Table.

### GEO database analysis

To investigate the differential gene expression in the liver tissues between *S*. *japonicum* infected and uninfected mice, the gene expression profiles of GSE14367 (6 wpi vs uninfected C57BL/6J mice, 7 wpi vs uninfected C57BL/6J mice) and GSE59276 (6 wpi vs uninfected C57BL/6J mice) were acquired and then assessed from the GEO database, an open database that documents high-throughput microarray empirical data [56]. P-value <0.01 and |log fold change (FC)| > 2 were used as the cut-off criteria to select the significant differentially expressed genes. The genes with logFC > 2 were thought to be upregulated genes, and those with logFC < −2 were regarded as downregulated genes.

### RNA extraction and mRNA/miRNA quantification

To evaluate the level of mRNAs and miRNAs in LX-2 cells and liver tissues, total RNA was extracted from LX-2 cells and liver tissues by TRIzol reagent (Invitrogen, USA) according to the manufacturer’s instructions [57] and quantified by Nanodrop (Thermo Scientific USA). For mRNAs, reverse-transcription was performed using a Hifair III 1st Strand cDNA Synthesis SuperMix for qPCR kit (Yeasen, China). The resulting cDNA was used as template for qPCR with Hieff qPCR SYBR Green Master Mix (Yeasen, China). The relative mRNA expression levels of genes were quantified with Gapdh served as an endogenous control. The LightCycler 96 system (Roche, China) was used for qPCR analysis. The cycling conditions were as follows: preincubation, 95°C for 60 s; 2 step amplification, 95°C for 5 s, and 60°C for 30 s, for 40 cycles; melting, 95°C for 10 s, 65°C for 60 s, 97°C for 1 s. For miRNAs, the first-strand cDNA was reverse-transcribed using the miRNA First Strand cDNA Synthesis kit (Stem-loop Method) (Sangon biotech, China) with a stem-loop RT primer designed by each miRNA. The resulting miRNA cDNA was used as template for qPCR with MicroRNAs qPCR Kit (SYBR Green Method) (Sangon biotech, China). The relative expression levels of miRNAs were quantified with U6 served as an endogenous control. The LightCycler 96 system (Roche, China) was used for qPCR analysis. The cycling conditions were as follows: preincubation, 95°C for 60 s; 2 step amplification, 95°C for 5 s, and 62°C for 30 s, for 40 cycles; melting, 95°C for 10 s, 65°C for 60 s, 97°C for 1 s. All samples were assessed in triplicate.

To evaluate the level of miRNAs in the serum, all miRNAs in serum from BALB/c mice were extracted using miRcute Serum/Plasma miRNA Isolation Kit (TIANGEN, China) according to the manufacturer’s recommendations. The procedures of first-strand cDNA reverse-transcription and qPCR for miRNAs were the same as above.

The 2^-ΔΔCt^ method [58] was used to calculate the fold change in the expression of all the mRNAs and miRNAs and all samples were assessed in triplicate. The primers used in this study are listed in S12 Table.

### Statistical analysis

Data were analyzed with SPSS 25.0 software (SPSS Inc., USA) and expressed as mean ± standard deviation (SD) of three independent biological replicates. Data were statistically analyzed with Student’s t-tests. A P-value of <0.05 was considered statistically significant in statistical analysis.

## Supporting information

S1 Fig Comparison of *Sj*-miRNAs coming from *S*. *japonicum* worm-derived, egg-derived EVs and primary HSCs of infected mice. (TIF)

S2 Fig TEM analysis of *Sj*EV-depleted ESPs and functional assays. (A) TEM image of *Sj*EV-depleted ESPs. Scale bar, 200 nm. (B) Relative expression of seven *Sj*-miRNAs after treatment of *Sj*EV-depleted ESPs, *Sj*EVs and dynasore (n = 3). (C) Treatment efficiency analysis after treated with NC or sja-let-7 inhibitor for 48 h (n = 3). All graph data are expressed as the mean ± SD of at least three biological replicates per group. *P< 0.05, **P< 0.01, ns, not significant.
(TIF)

S3 Fig Col1α2 is the target gene of sja-let-7. (A) Venn diagram showing 1167 potential target genes overlap in miRanda and RNAhybrid database. (B) GO analysis of 1167 potential target genes. Col1α2 is identified from 3 GO terms, “collagen trimer”, “extracellular matrix” and “extracellular matrix part”. (C) Alignments of multiple let-7 family members. (D) Venn diagram showed that Col1α2 was only a target gene of sja-let-7 and was not included in the target genes of the host let-7 family. (E) Schematic diagram of transwell systems composed by worms coming from KM mice with treatment of NC or sja-let-7 inhibitor. (F) Detection of Col1α2 mRNA expression of the LX-2 cells (n = 3). (G) Schematic diagram of transwell systems composed by worms coming from BALB\c mice with treatment of NC or sja-let-7 inhibitor. (H) Detection of Col1α2 mRNA expression of the LX-2 cells (n = 3). (I) Schematic diagram of *Sj*EVs incubated with LX-2 with treatment of NC or sja-let-7 inhibitor. (J) Detection of Col1α2 mRNA expression of the LX-2 cells (n = 3). All graph data are expressed as the mean ± SD of at least three biological replicates per group. *P< 0.05, **P< 0.01, ns, not significant. Panel E, G and I was created with Biorender.com.
(TIF)

S4 Fig Observation of the overall course of liver fibrosis in BALB/c mice during 0–12 wpi. (A) Liver and spleen appearance, hematological index of mice. (B-C) Hematological lym% and Neu% index of mice (n = 3). (D-E) Liver and spleen indexes of mice (n = 3). (F) Detection of hydroxyproline content in the liver tissues (n = 3). All graph data are expressed as the mean ± SD of at least three biological replicates per group. *P< 0.05, **P< 0.01, ns, not significant.
(TIF)

S5 Fig Establishment of schistosome-induced liver fibrosis in BALB/c mice. (A) Liver histological analysis through H&E, Masson and Sirius red staining during 0–12 wpi. Black arrows indicate the egg and the egg granuloma. Scale bar, 200 μm. *Insets* show a higher magnification of the outlined area. Scale bar, 100 μm. (B-F) Detection of α-SMA, Col1α1, Col3α1, Col1α2 and sja-let-7 relative expression in the mice liver during 0–12 wpi (n = 3). All graph data are expressed as the mean ± SD of at least three biological replicates per group. *P< 0.05, **P< 0.01, ns, not significant.
(TIF)

S6 Fig Volcano plots showing GEO database of C57BL/6J mice liver fibrosis. (TIF)

S7 Fig Sja-let-7 attenuates the fibrotic progression of schistosome-induced liver fibrosis. (A) Liver and spleen appearance. (B-C) Liver and spleen indexes of mice (n = 6). (D) Hematological index. (E-G) Hematological WBC, lym% and Neu% index of mice (n = 6). (H-J) Detection of AST, ALT and hydroxyproline content in the liver tissues (n = 6). (K-M) α-SMA, Col1α1 and Col1α3 positive area of IHC analysis (n = 6). (N-P) α-SMA, Col1α1 and Col1α3 positive area of immunofluorescence analysis (n = 6). (Q) ELISA of circulating α-SMA level (n = 6). (R-U) Detection of IL-1β, IL-6, TNF-α and HMGB1 mRNA expression (n = 6).
(TIF)

S8 Fig Establishment of transwell systems with treatment of NC or sja-let-7 inhibitor. This figure was created with Biorender.com.
(TIF)

S9 Fig The workflow of *Sj*EVs isolation procedure. This figure was created with Biorender.com.
(TIF)

S10 Fig Distribution of liver, spleen tissues and blood sample of each mouse. (TIF)

S1 Table Comparison of *Sj*-miRNAs coming from *S*. *japonicum* worm-derived, egg-derived EVs and primary HSCs of infected mice. (XLSX)

S2 Table *S*. *japonicum* proteins identified in EVs by LC-MS/MS. (XLSX)

S3 Table Schistosome EV-associated proteins selected from all EVs proteins identified by LC-MS/MS. (XLSX)

S4 Table Potential target genes of sja-let-7 identified by RNAhybrid and miRanda. (XLSX)

S5 Table GO enrichment analysis of 1167 target genes of sja-let-7 identified by RNAhybird and miRanda. (XLSX)

S6 Table Target genes of *M*. *musculus* let-7 family identified by RNAhybrid and miRanda. (XLSX)

S7 Table Cross-reference of target genes of sja-let-7 and *M*. *musculus* let-7 family. (XLSX)

S8 Table GO functional analysis of target genes of sja-let-7. (XLSX)

S9 Table The sequence of miRNA mimics, inhibitor and agomir. (DOCX)

S10 Table Antibodies used in the experiment. (DOCX)

S11 Table Probes used in the FISH analysis. (DOCX)

S12 Table Primers used in the experiment. (DOCX)

S1 Data Details for *Sj*EVs collection and function analysis. (DOCX)

S2 Data Excel spreadsheet containing, in separate sheets, the underlying numerical data and statistical analysis for Figures. (XLSX)
