# Supplementary figures and images for: Sja-let-7 suppresses the development of liver fibrosis via Schistosoma japonicum extracellular vesicles
Source: PLoS Pathog. 2024 Apr 10;20(4):e1012153. doi: 10.1371/journal.ppat.1012153 (PMC11034668; doi:10.1371/journal.ppat.1012153)

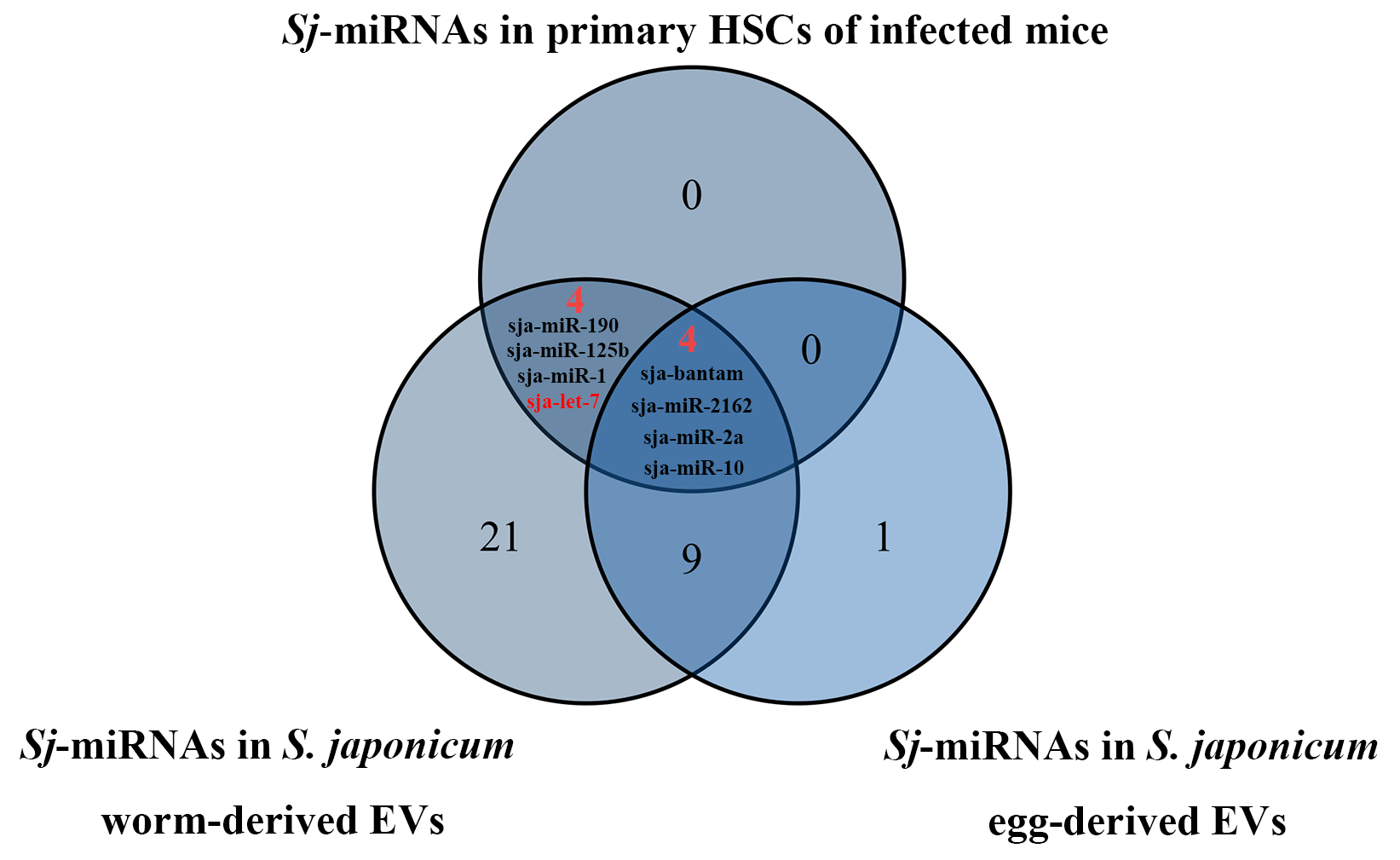

Supplement: S1 Fig — (TIF) [file ppat.1012153.s001.tif]

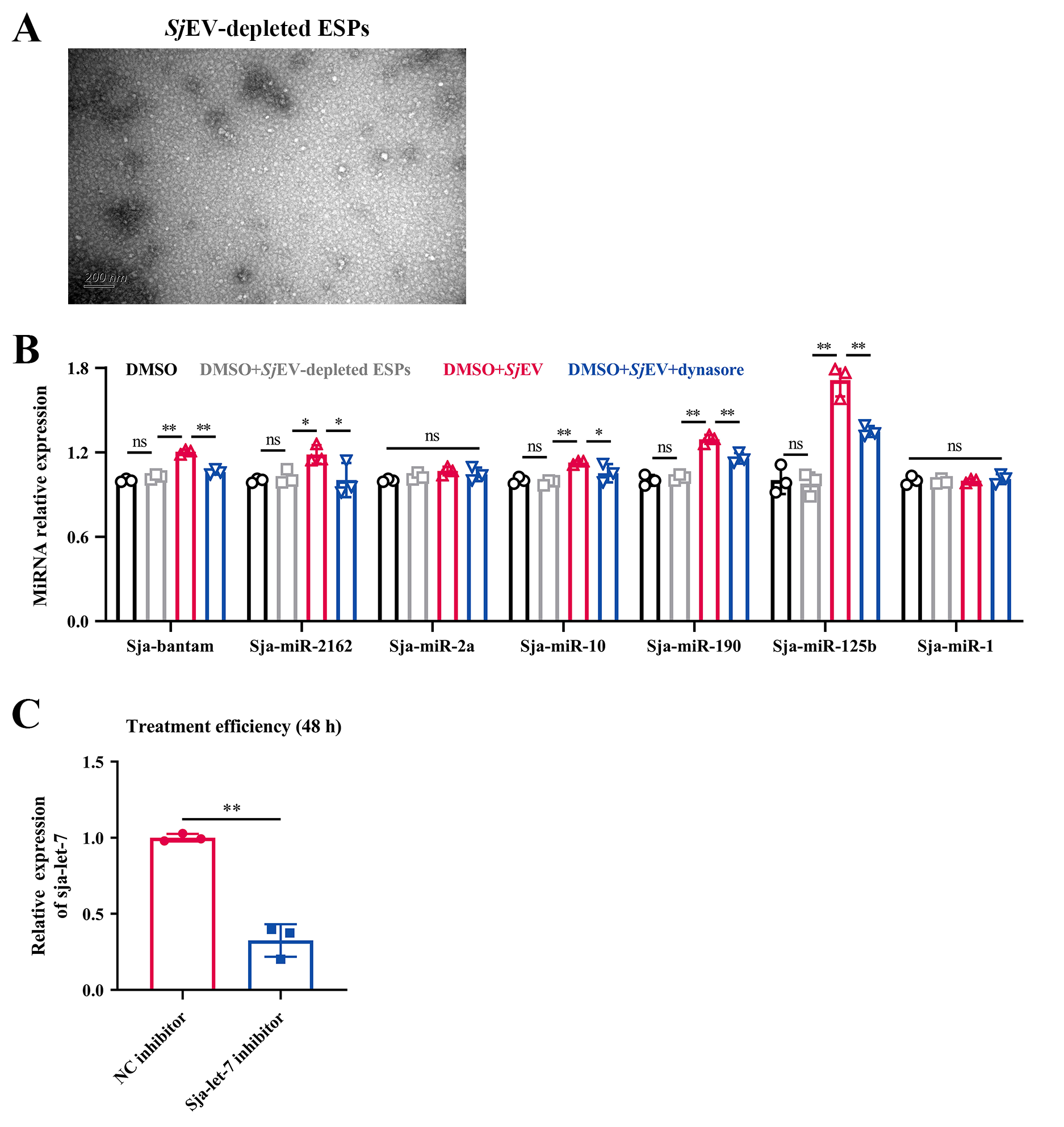

Supplement: S2 Fig — (A) TEM image of SjEV-depleted ESPs. Scale bar, 200 nm. (B) Relative expression of seven Sj-miRNAs after treatment of SjEV-depleted ESPs, SjEVs and dynasore (n = 3). (C) Treatment efficiency analysis after treated with NC or sja-let-7 inhibitor for 48 h (n = 3). All graph data are expressed as the mean ± SD of at least three biological replicates per group. *P< 0.05, **P< 0.01, ns, not significant. (TIF) [file ppat.1012153.s002.tif]

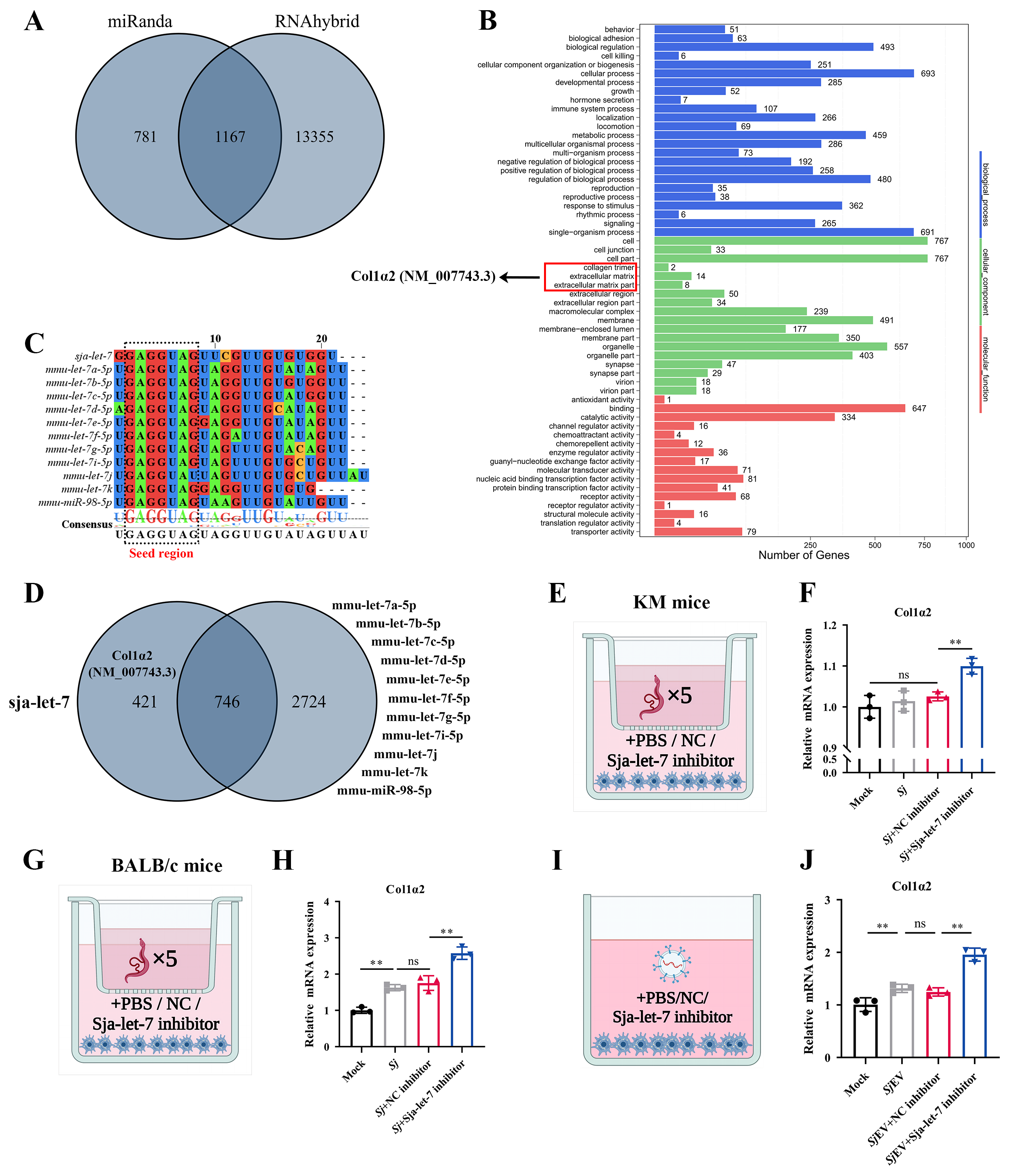

Supplement: S3 Fig — (A) Venn diagram showing 1167 potential target genes overlap in miRanda and RNAhybrid database. (B) GO analysis of 1167 potential target genes. Col1α2 is identified from 3 GO terms, “collagen trimer”, “extracellular matrix” and “extracellular matrix part”. (C) Alignments of multiple let-7 family members. (D) Venn diagram showed that Col1α2 was only a target gene of sja-let-7 and was not included in the target genes of the host let-7 family. (E) Schematic diagram of transwell systems composed by worms coming from KM mice with treatment of NC or sja-let-7 inhibitor. (F) Detection of Col1α2 mRNA expression of the LX-2 cells (n = 3). (G) Schematic diagram of transwell systems composed by worms coming from BALB\c mice with treatment of NC or sja-let-7 inhibitor. (H) Detection of Col1α2 mRNA expression of the LX-2 cells (n = 3). (I) Schematic diagram of SjEVs incubated with LX-2 with treatment of NC or sja-let-7 inhibitor. (J) Detection of Col1α2 mRNA expression of the LX-2 cells (n = 3). All graph data are expressed as the mean ± SD of at least three biological replicates per group. *P< 0.05, **P< 0.01, ns, not significant. Panel E, G and I was created with Biorender.com. (TIF) [file ppat.1012153.s003.tif]

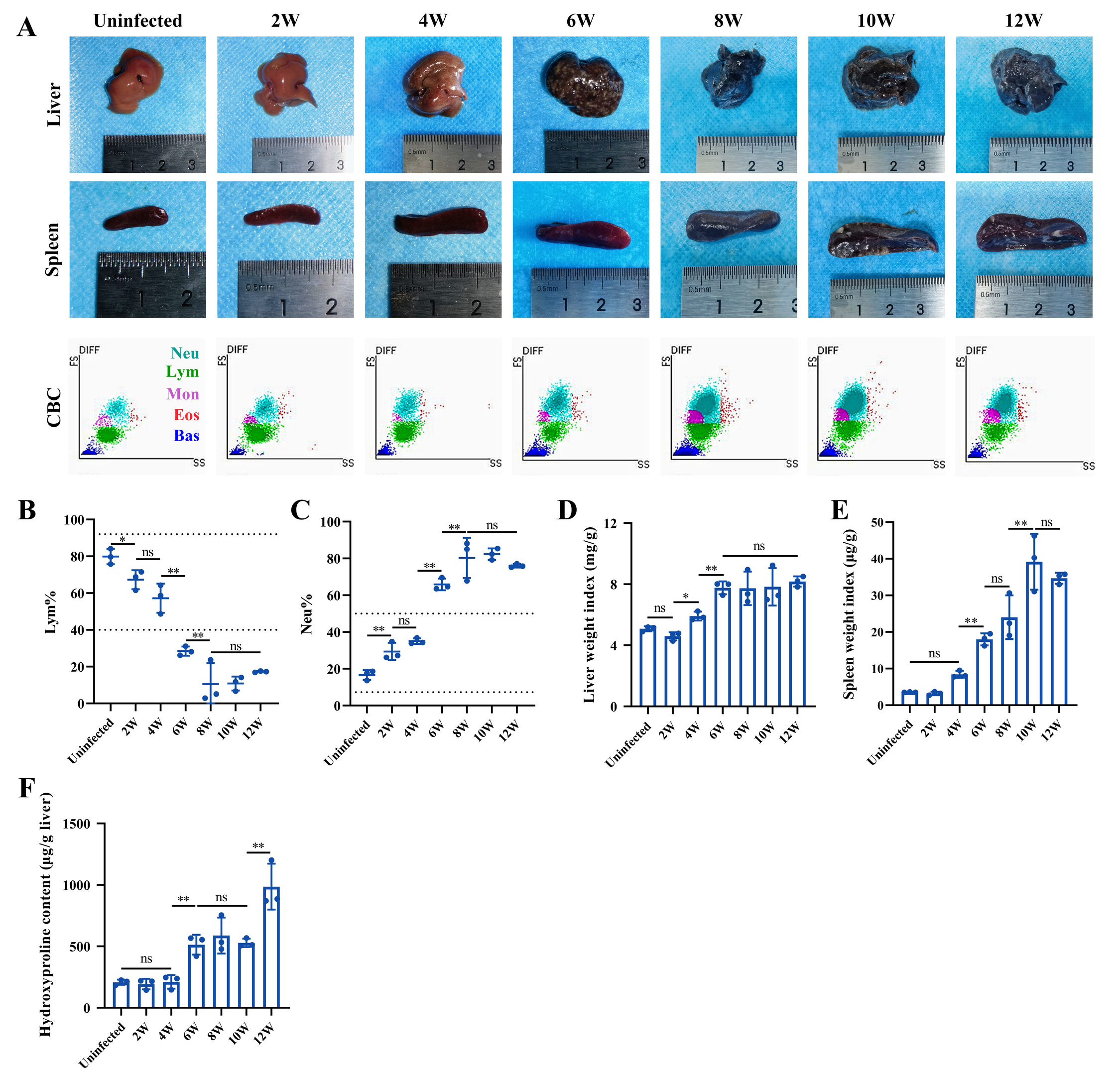

Supplement: S4 Fig — (A) Liver and spleen appearance, hematological index of mice. (B-C) Hematological lym% and Neu% index of mice (n = 3). (D-E) Liver and spleen indexes of mice (n = 3). (F) Detection of hydroxyproline content in the liver tissues (n = 3). All graph data are expressed as the mean ± SD of at least three biological replicates per group. *P< 0.05, **P< 0.01, ns, not significant. (TIF) [file ppat.1012153.s004.tif]

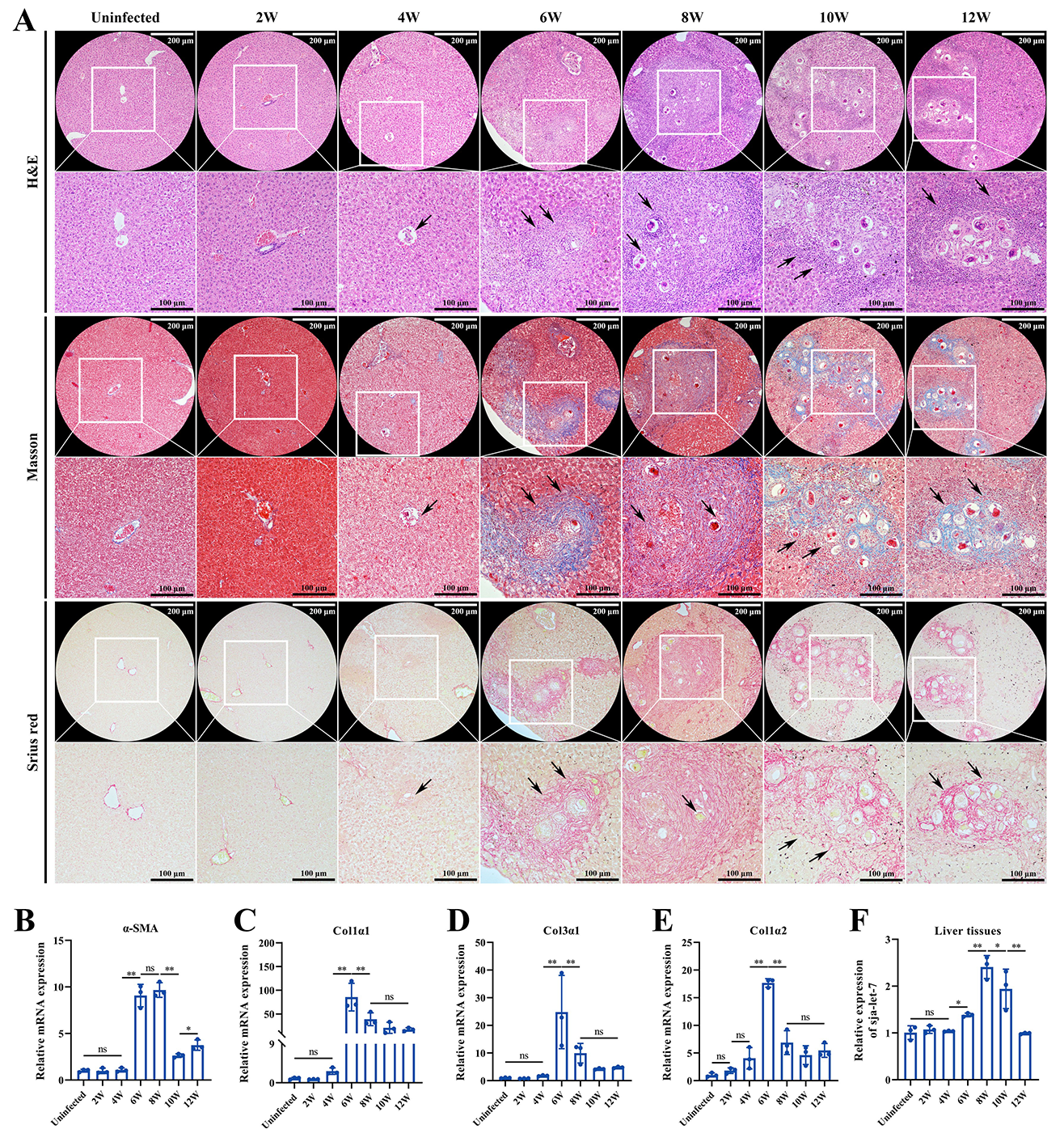

Supplement: S5 Fig — (A) Liver histological analysis through H&E, Masson and Sirius red staining during 0–12 wpi. Black arrows indicate the egg and the egg granuloma. Scale bar, 200 μm. Insets show a higher magnification of the outlined area. Scale bar, 100 μm. (B-F) Detection of α-SMA, Col1α1, Col3α1, Col1α2 and sja-let-7 relative expression in the mice liver during 0–12 wpi (n = 3). All graph data are expressed as the mean ± SD of at least three biological replicates per group. *P< 0.05, **P< 0.01, ns, not significant. (TIF) [file ppat.1012153.s005.tif]

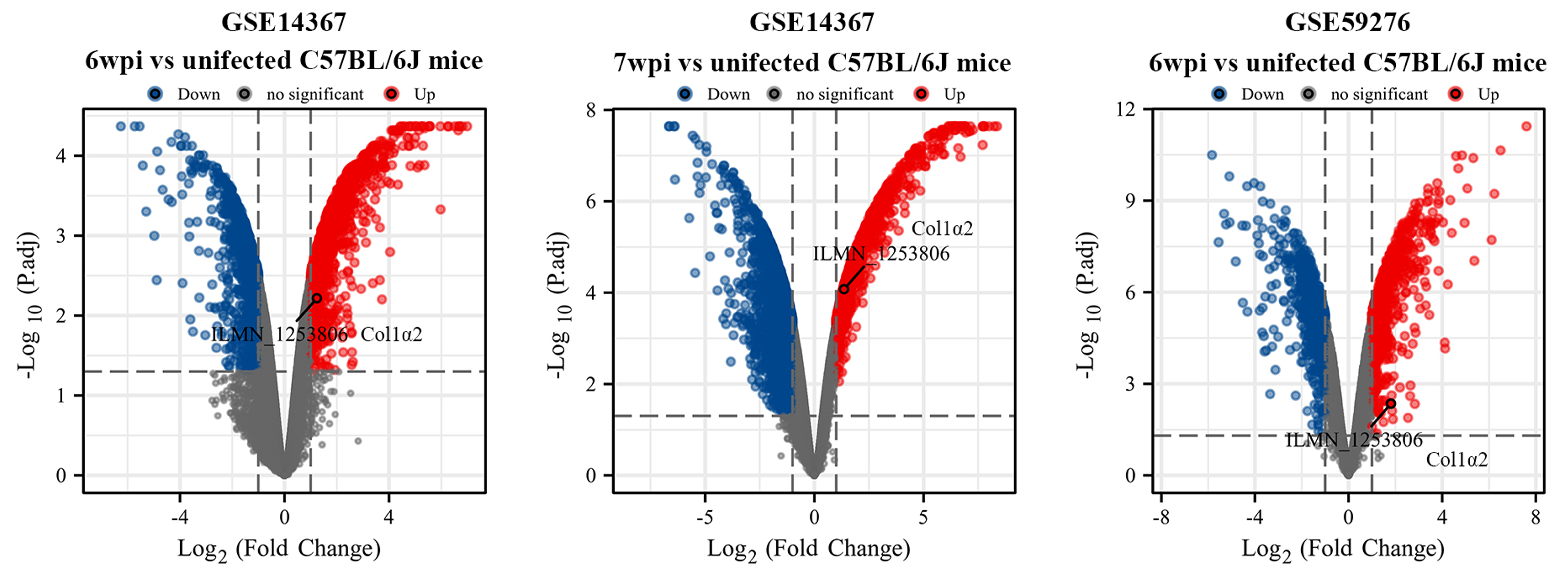

Supplement: S6 Fig — (TIF) [file ppat.1012153.s006.tif]

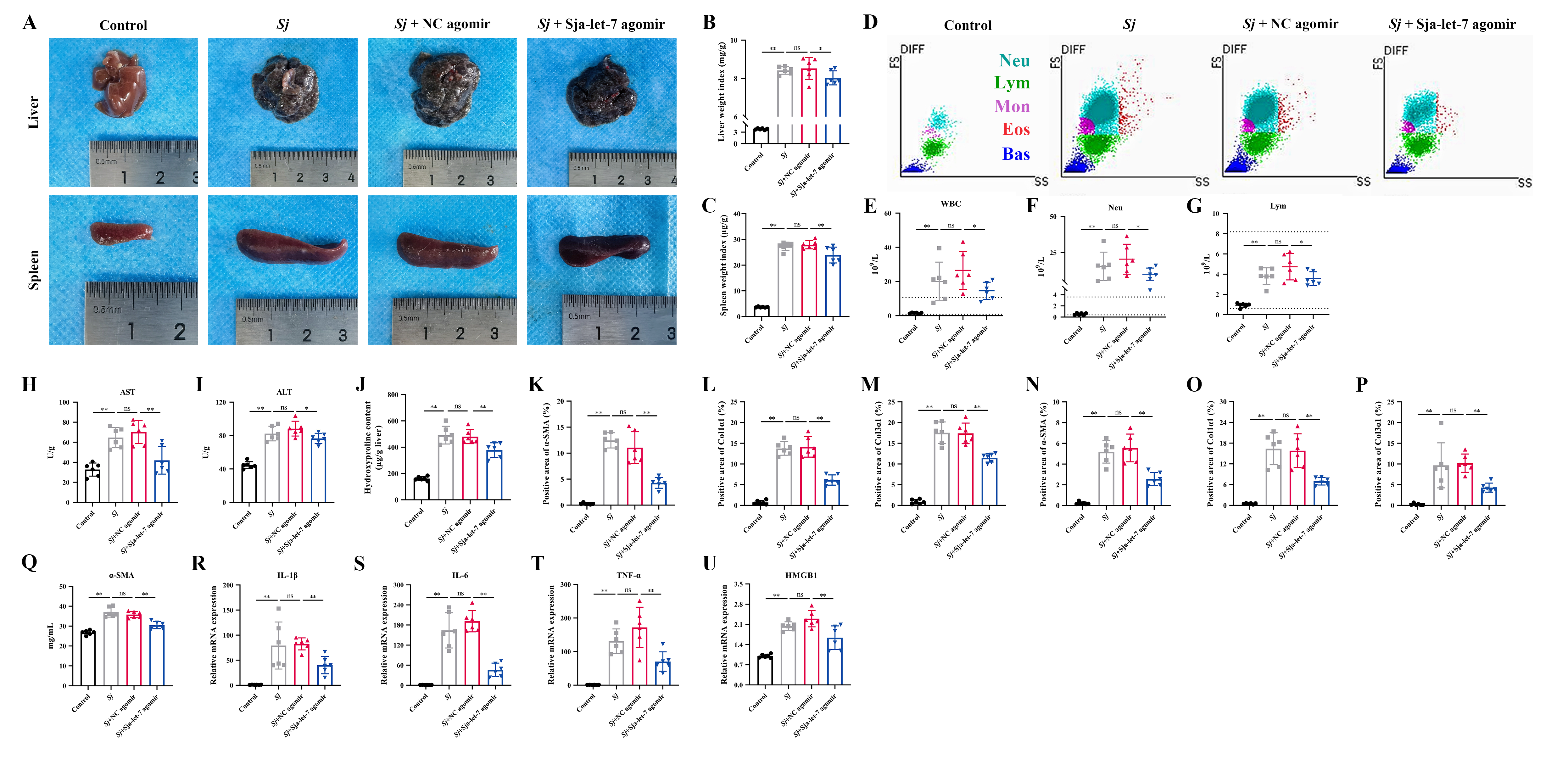

Supplement: S7 Fig — (A) Liver and spleen appearance. (B-C) Liver and spleen indexes of mice (n = 6). (D) Hematological index. (E-G) Hematological WBC, lym% and Neu% index of mice (n = 6). (H-J) Detection of AST, ALT and hydroxyproline content in the liver tissues (n = 6). (K-M) α-SMA, Col1α1 and Col1α3 positive area of IHC analysis (n = 6). (N-P) α-SMA, Col1α1 and Col1α3 positive area of immunofluorescence analysis (n = 6). (Q) ELISA of circulating α-SMA level (n = 6). (R-U) Detection of IL-1β, IL-6, TNF-α and HMGB1 mRNA expression (n = 6). (TIF) [file ppat.1012153.s007.tif]

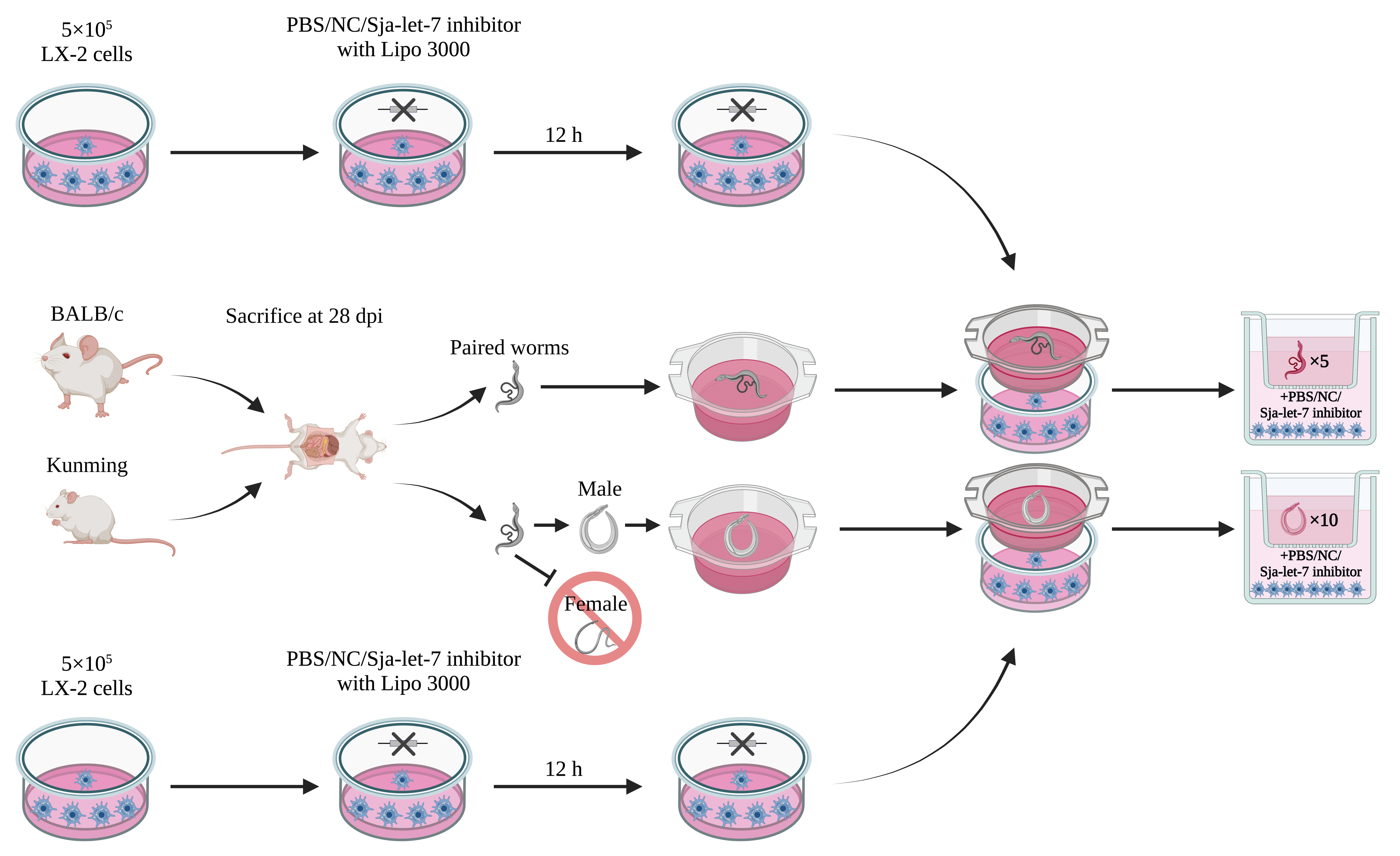

Supplement: S8 Fig — This figure was created with Biorender.com. (TIF) [file ppat.1012153.s008.tif]

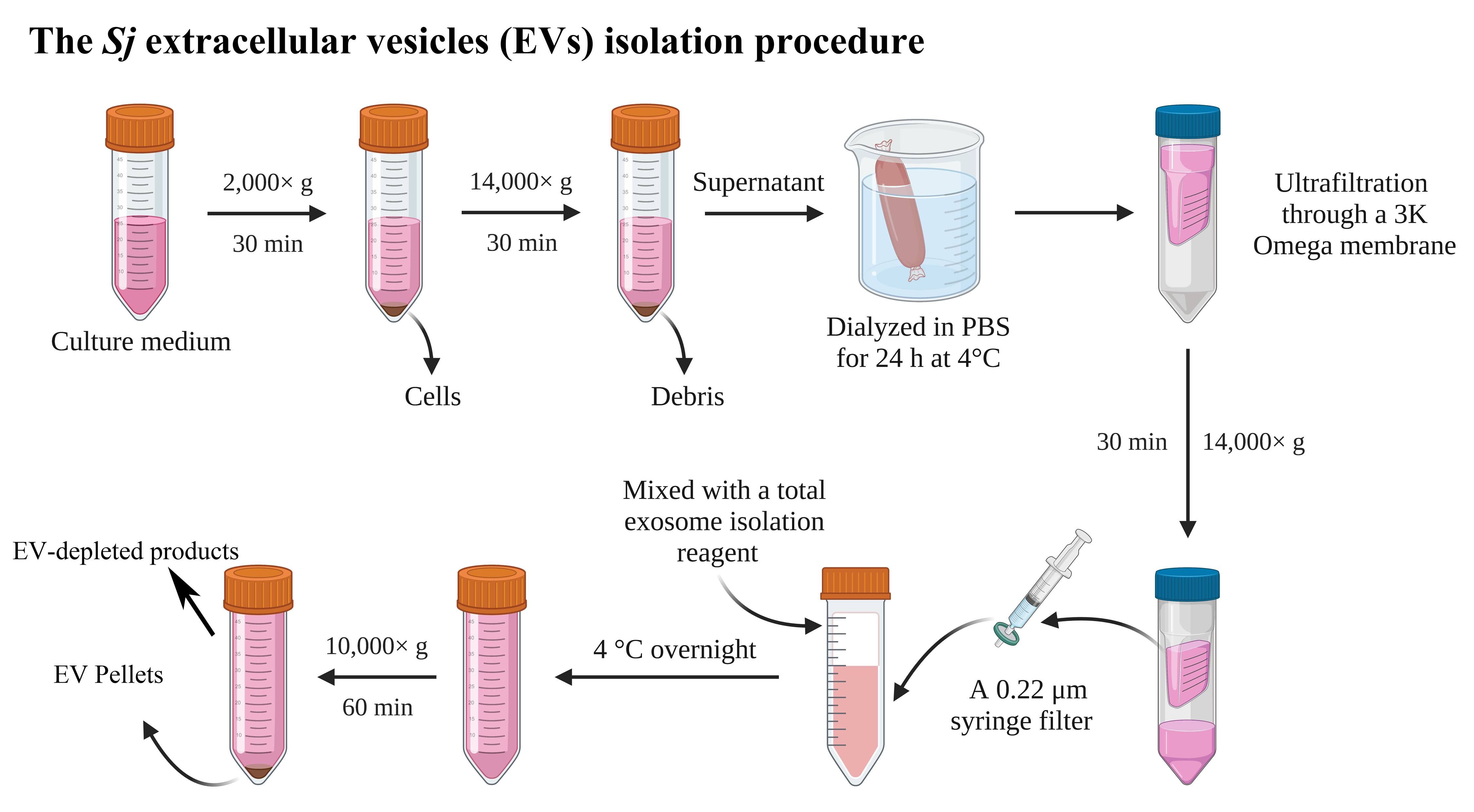

Supplement: S9 Fig — This figure was created with Biorender.com. (TIF) [file ppat.1012153.s009.tif]

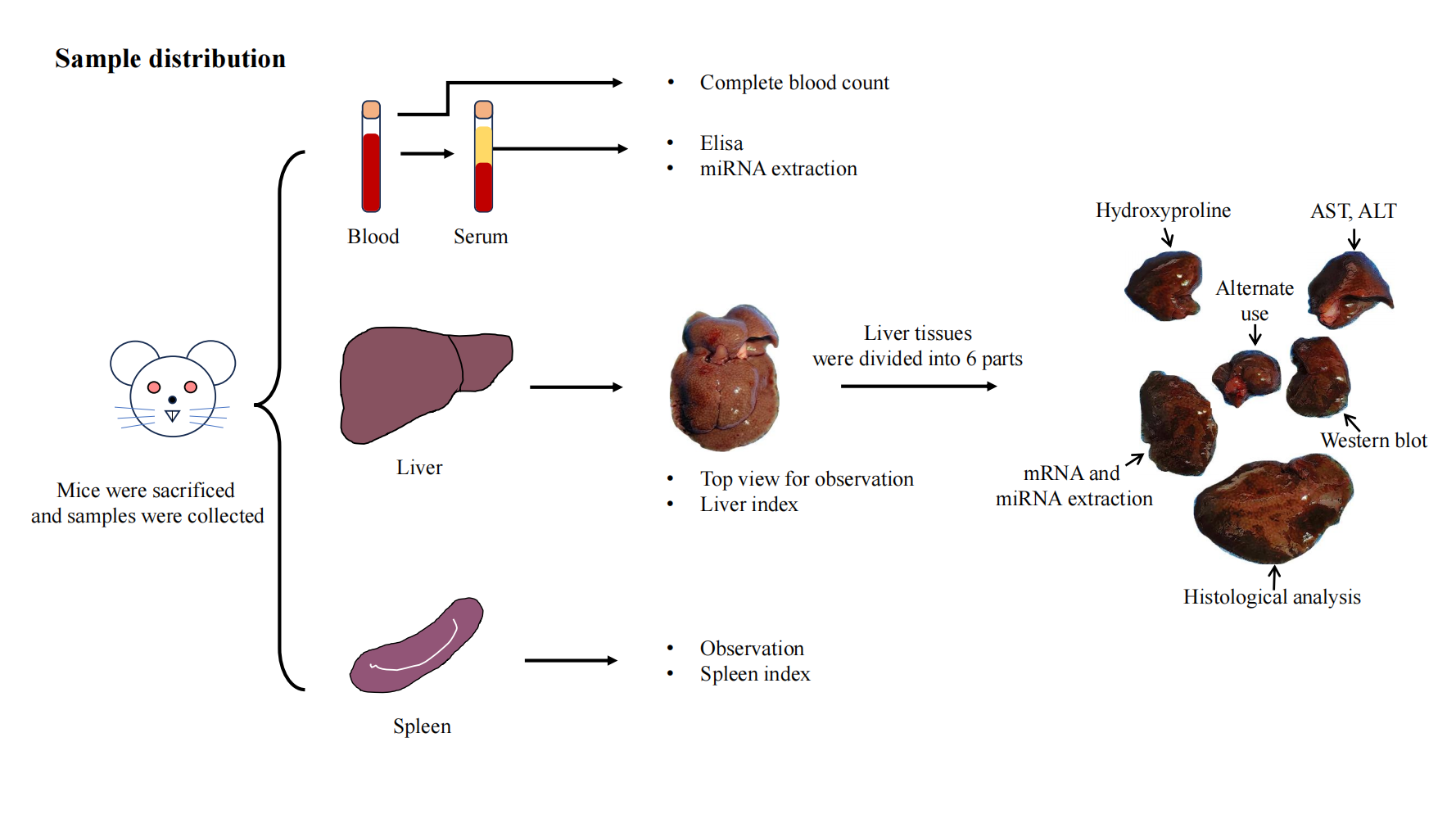

Supplement: S10 Fig — (TIF) [file ppat.1012153.s010.tif]
