# Supplementary material for: Sja-let-7 suppresses the development of liver fibrosis via Schistosoma japonicum extracellular vesicles
Source: PLoS Pathog. 2024 Apr 10;20(4):e1012153. doi: 10.1371/journal.ppat.1012153 (PMC11034668; doi:10.1371/journal.ppat.1012153)
Supplement: S9 Table — (DOCX) [file ppat.1012153.s019.docx]

S9 Table. The sequence of miRNA mimics, inhibitor and agomir.

| Name | Sense sequence (5'-3') | Anti-sense sequence (5'-3') |
| --- | --- | --- |
| Sja-let-7 inhibitor | ACCACACAACGAACUACCUCC |  |
| NC inhibitor | CAGUACUUUUGUGUAGUACAA |  |
| Sja-let-7 agomir | GGAGGUAGUUCGUUGUGUGGU | CACACAACGAACUACCUCCUU |
| NC agomir | UUCUCCGAACGUGUCACGUTT | ACGUGACACGUUCGGAGAATT |
| Sja-let-7 mimics | GGAGGUAGUUCGUUGUGUGGU | CACACAACGAACUACCUCCUU |
| NC mimics | UUCUCCGAACGUGUCACGUTT | ACGUGACACGUUCGGAGAATT |
