# Supplementary material for: Sja-let-7 suppresses the development of liver fibrosis via Schistosoma japonicum extracellular vesicles
Source: PLoS Pathog. 2024 Apr 10;20(4):e1012153. doi: 10.1371/journal.ppat.1012153 (PMC11034668; doi:10.1371/journal.ppat.1012153)
Supplement: S10 Table — (DOCX) [file ppat.1012153.s020.docx]

S10 Table . Antibodies used in the experiment

| Experiments | Primary antibody | Source  (Catalogue No.) | Host | Working conditions | Secondary antibody | Source (Catalogue No.) | Working conditions |
| --- | --- | --- | --- | --- | --- | --- | --- |
| Immunohistochemical assay | TGF-β1 | Servicebio  (GB11179) | Rabbit | 1:500 | HRP conjugated Goat Anti-Rabbit IgG (H+L) | Servicebio  (GB23303) | 1:200 |
|  | p-SMAD2/3 | SAB  (12241) | Rabbit | 1:200 |  |  |  |
|  | α-SMA | Servicebio  (GB111364) | Rabbit | 1:2000 |  |  |  |
|  | Col1α1 | Servicebio  (GB11022) | Rabbit | 1:1000 |  |  |  |
|  | Col1α2 | Proteintech  (14695-1-AP) | Rabbit | 1:500 |  |  |  |
|  | Col3α1 | Servicebio  (GB111629) | Rabbit | 1:500 |  |  |  |
| Immunofluorescence analysis | α-SMA | Servicebio  (GB13044) | Mouse | 1:1000 | Cy5 conjugated Goat Anti-mouse IgG (H+L) | Servicebio  (GB27301) | 1:400 |
|  | Col1α1 | Servicebio  (GB11022) | Rabbit | 1:3000 | Cy3 conjugated Goat Anti-Rabbit IgG (H+L) | Servicebio  (GB21303) | 1:300 |
|  | Col3α1 | Servicebio  (GB111629) | Rabbit | 1:200 | Alexa Fluor® 488-conjugated Goat Anti-Rabbit IgG (H+L) | Servicebio  (GB25303) | 1:400 |
