# Supplementary material for: Sja-let-7 suppresses the development of liver fibrosis via Schistosoma japonicum extracellular vesicles
Source: PLoS Pathog. 2024 Apr 10;20(4):e1012153. doi: 10.1371/journal.ppat.1012153 (PMC11034668; doi:10.1371/journal.ppat.1012153)
Supplement: S11 Table — (DOCX) [file ppat.1012153.s021.docx]

S11 Table. Probes used in the FISH analysis.

| Probe name | Sequence |
| --- | --- |
| sja-let-7 | 5’-ACCACACAACGAACTACCTCC-3’ |
| Col1α2 | 5’-TGTCTTGCCCCATTCATTTGTCTTTTT-3’ |
|  | 5’-CAGGCGAGATGGCTTATTTGTTTTGT-3’ |
|  | 5’-GGCATGTTGCTAGGCACGAAGTTACT-3’ |
