# Supplementary material for: Sja-let-7 suppresses the development of liver fibrosis via Schistosoma japonicum extracellular vesicles
Source: PLoS Pathog. 2024 Apr 10;20(4):e1012153. doi: 10.1371/journal.ppat.1012153 (PMC11034668; doi:10.1371/journal.ppat.1012153)
Supplement: S12 Table — (DOCX) [file ppat.1012153.s022.docx]

S12 Table. Primers used in the experiment

| Primer | Sequences (5’-3’) |
| --- | --- |
| Human-GAPDH-F | GTCTCCTCTGACTTCAACAGCG |
| Human-GAPDH-R | ACCACCCTGTTGCTGTAGCCAA |
| Human-α-SMA-F | ATGCTTCTAAAACACTTTCCTGCTC |
| Human-α-SMA-R | AGCTTTGGCTAGGAATGATTTGG |
| Human-Col1α1-F | GGTTCGGAGGAGAGTCAGGAAG |
| Human-Col1α1-R | TTTCAGCAACACAGTTACACAAGG |
| Human-Col1α2-F | CTGGCACCACACCTTCTACAATG |
| Human-Col1α2-R | AATGTCACGCACGATTTCCCGC |
| Human-Col3α1-F | TGGTCTGCAAGGAATGCCTGGA |
| Human-Col3α1-R | TCTTTCCCTGGGACACCATCAG |
| Human-Smad2-F | TGCCACGGTAGAAATGACAAGAAGG |
| Human-Smad2-R | GGGTGCCAGCCATATCTCTGATTAC |
| Human-Smad3-F | GGAGCGGAGTACAGGAGACAGAC |
| Human-Smad3-R | CTAAGACACACTGGAACAGCGGATG |
| Human-Smad7-F | CTCGGAAGTCAAGAGGCTGTGTTG |
| Human-Smad7-R | TCTAGTTCGCAGAGTCGGCTAAGG |
| Mouse-GAPDH-F | AACGGGAAGCCCATCACCATC |
| Mouse-GAPDH-R | AAGACACCAGTAGACTCCACGA |
| Mouse-IL-1β-F | ATGAAAGACGGCACACCCAC |
| Mouse-IL-1β-R | GCTTGTGCTCTGCTTGTGAG |
| Mouse-IL-6-F | TGCAAGAGACTTCCATCCAGT |
| Mouse-IL-6-R | GTGAAGTAGGGAAGGCCG |
| Mouse-TNFα-F | CAGCCGATGGGTTGTACCTT |
| Mouse-TNFα-R | TGTGGGTGAGGAGCACGTAGT |
| Mouse-HMGB1-F | GGCGAGCATCCTGGCTTATC |
| Mouse-HMGB1-R | GGCTGCTTGTCATCTGCTG |
| Mouse-α-SMA-F | TCAGCGCCTCCAGTTCCT |
| Mouse-α-SMA-R | AAAAAAAACCACGAGTAACAAATCAA |
| Mouse-Col1α1-F | ACGTCCTGGTGAAGTTGGTC |
| Mouse-Col1α1-R | CAGGGAAGCCTCTTTCTCCT |
| Mouse-Col1α2-F | CCAGGGCTGTTTTCCCATCC |
| Mouse-Col1α2-R | GCTCTGTGCTTCGTCACCCA |
| Mouse-Col3α1-F | GCCCACAGCCTTCTACACCT |
| Mouse-Col3α1-R | GCCAGGGTCACCATTTCTC |
| Mouse-TGF-β1-F | ATTCCTGGCGTTACCTTGG |
| Mouse-TGF-β1-R | AGCCCTGTATTCCGTCTCCT |
| Mouse-Smad2-F | GTGGCATACTGGGAGGAGAA |
| Mouse-Smad2-R | TTGTTGTCCGAATTGAGCTG |
| Mouse-Smad3-F | GAGACATTCCACGCTTCACA |
| Mouse-Smad3-R | GCTGCATTCCGGTTAACATT |
| Mouse-Smad7-F | GTGTTGCTGTGAATCTTACG |
| Mouse-Smad7-R | AGAAGAAGTTGGGAATCTGA |
| common-REVERSE | CAGTGCAGGGTCCGAGGT |
| mouse U6-RT-primer | GTCGTATCCAGTGCAGGGTCCGAGGTATTCGCACTGGATACGACAAAAAT |
| mouse U6-FORWARD | GAAGATTTAGCATGGCCCCTGC |
| sja-bantam-RT-primer | GTCGTATCCAGTGCAGGGTCCGAGGTATTCGCACTGGATACGACACCAGC |
| sja-bantam-FORWARD | AGCAGGTGAGATCGCGATTAA |
| sja-let-7-RT-primer | GTCGTATCCAGTGCAGGGTCCGAGGTATTCGCACTGGATACGACACCACA |
| sja-let-7-FORWARD | ACAACAACGGAGGTAGTTCGT |
| sja-miR-1-RT-primer | GTCGTATCCAGTGCAGGGTCCGAGGTATTCGCACTGGATACGACGACCAT |
| sja-miR-1-FORWARD | AAGGTTGTTGGAATGTGGCGAAG |
| sja-miR-1b-RT-primer | GTCGTATCCAGTGCAGGGTCCGAGGTATTCGCACTGGATACGACGCACAT |
| sja-miR-1b-FORWARD | CCACGGAGTGGAATGTTGTGAAG |
| sja-miR-2a-RT-primer | GTCGTATCCAGTGCAGGGTCCGAGGTATTCGCACTGGATACGACCGTTCA |
| sja-miR-2a-FORWARD | AAGCGCCTTCACAGCCAGTATT |
| sja-miR-10-RT-primer | GTCGTATCCAGTGCAGGGTCCGAGGTATTCGCACTGGATACGACCCAAAC |
| sja-miR-10-FORWARD | AGCAACTTAACCCTGTAGACCCG |
| sja-miR-61-RT-primer | GTCGTATCCAGTGCAGGGTCCGAGGTATTCGCACTGGATACGACGAAGTG |
| sja-miR-61-FORWARD | AACACGTGTGACTAGAAAGTGCAC |
| sja-miR-71a-RT-primer | GTCGTATCCAGTGCAGGGTCCGAGGTATTCGCACTGGATACGACTCTCAC |
| sja-miR-71a-FORWARD | CGACGCTTGAAAGACGATGG |
| sja-miR-71b-RT-primer | GTCGTATCCAGTGCAGGGTCCGAGGTATTCGCACTGGATACGACCGTCTC |
| sja-miR-71b-FORWARD | GCGCGTGAAAGACTTGAGTAGT |
| sja-miR-125b-RT-primer | GTCGTATCCAGTGCAGGGTCCGAGGTATTCGCACTGGATACGACGAGCAA |
| sja-miR-125b -FORWARD | AAGCGACCTCCCTGAGACTGAT |
| sja-miR-190-RT-primer | GTCGTATCCAGTGCAGGGTCCGAGGTATTCGCACTGGATACGACCACCAA |
| sja-miR-190-FORWARD | GCGCGTGATATGTATGGGTTAC |
| sja-miR-277-RT-primer | GTCGTATCCAGTGCAGGGTCCGAGGTATTCGCACTGGATACGACACGGGC |
| sja-miR-277-FORWARD | GCGCGTAAATGCATTTTCTG |
| sja-miR-277b-RT-primer | GTCGTATCCAGTGCAGGGTCCGAGGTATTCGCACTGGATACGACTCTAGG |
| sja-miR-277b-FORWARD | GCGCGAAAATGCATCATCTAC |
| sja-miR-750-RT-primer | GTCGTATCCAGTGCAGGGTCCGAGGTATTCGCACTGGATACGACAGTTGG |
| sja-miR-750-FORWARD | ACTTCCTCCCAGATCTGTCGCT |
| sja-miR-2162-RT-primer | GTCGTATCCAGTGCAGGGTCCGAGGTATTCGCACTGGATACGACAGAGTG |
| sja-miR-2162-FORWARD | CCTCGAGCTATTATGCAACGTT |
