# Supplementary material for: Sja-let-7 suppresses the development of liver fibrosis via Schistosoma japonicum extracellular vesicles
Source: PLoS Pathog. 2024 Apr 10;20(4):e1012153. doi: 10.1371/journal.ppat.1012153 (PMC11034668; doi:10.1371/journal.ppat.1012153)
Supplement: S1 Data — (DOCX) [file ppat.1012153.s023.docx]

**S1 Data. Details for *Sj*EVs collection and function analysis**

| Titles | Details |
| --- | --- |
| Helminths | |
| Helminth species | *Schistosoma japonicum* Anhui strain |
| Lifestage | 28 days |
| Number of worms | ~15 worm pairs per mL of media |
| Washing buffer | Phosphate buffer solution (PBS, Corning, USA) |
| Host |  |
| Host species | New Zealand rabbits |
| Host age and sex | 7–8 weeks old; male |
| Host tissue from where the worms were isolated | Hepatic portal vein |
| Helminth cultivation |  |
| Culture length | 2 hours |
| Composition of culture media | RPMI-1640 (Corning, USA) containing 1% penicillin-streptomycin (Thermo Fisher Scientific, USA) |
| Number of worms per mL of media | 15 worm pairs |
| Incubation temperature and % CO2 | 37°C, 5% CO_2_ |
| Parasite viability | Visual inspection |
| Number of parasite pre-incubation washes | 3–5 times with 50 mL PBS |
| EV separation and QC |  |
| ES collection timepoints | Every 2 hours during cultivation |
| EV storage details | -80 °C |
| EV separation technique used and details | After 2 h incubation, the supernatant was collected and fresh culture medium was added for the next collection (the whole collecting procedure could last for 3–4 days until the worms were less active). The pellets in the collected supernatant were discarded by centrifugation at 2,000× g and 14,000× g for 30 min each at 4°C, respectively. Then, the supernatant was collected and dialyzed in PBS for 24 h at 4°C followed by centrifugal ultrafiltration through a 3K Omega membrane (Pall, USA). The supernatant was then filtered using a 0.22 μm syringe filter (Pall, USA) and a total EV isolation kit (Thermo Fisher Scientific, USA) was used according to the manufacturer’s instructions. The EV pellet was resuspended in 200 μL of PBS and then stored at -80 °C until further analysis. |
| Details of low speed differential centrifugation to pre-clear ES of eggs and debris | To pre-clear ES of eggs and debris. The pellets in the collected supernatant were discarded by centrifugation at 2,000× g and 14,000× g for 30 min each at 4°C, respectively. |
| Functional studies of helminth EVS |  |
| Measurement of number of particles | 9.5×10^11^/ mL |
| Size distribution | 124.8±58.0 nm |
| EV labelling method | PKH67 labelling (Sigma Aldrich, USA) |
| Controls of EV labelling | *Sj*EV-depleted ESPs (Excretory-secretory products) |
| Controls used for functional studies and how they were obtained | *Sj*EV-depleted ESPs (Excretory-secretory products), the supernatant after pelleting the *Sj*EVs was collected |
| Normalisation of EVs for functional studies (number of vesicles/mL) | 4.3×10^10^ particles/mL |
